# Human gut microbiota is associated with HIV-reactive immunoglobulin at baseline and following HIV vaccination

**DOI:** 10.1371/journal.pone.0225622

**Published:** 2019-12-23

**Authors:** Jacob A. Cram, Andrew J. Fiore-Gartland, Sujatha Srinivasan, Shelly Karuna, Giuseppe Pantaleo, Georgia D. Tomaras, David N. Fredricks, James G. Kublin

**Affiliations:** 1 Vaccine and Infectious Disease Division, Fred Hutchinson Cancer Research Center, Seattle, Washington, United States of America; 2 University of Maryland Center for Environmental Science, Cambridge, Maryland, United States of America; 3 HIV Vaccine Trials Network, Vaccine and Infectious Disease Division, Fred Hutchinson Cancer Research Center, Seattle, Washington, United States of America; 4 Service of Immunology and Allergy, and Swiss Vaccine Research Institute, Lausanne University Hospital (CHUV), Lausanne, Switzerland; 5 Duke Human Vaccine Institute, Duke University School of Medicine, Durham, North Carolina, United States of America; Northwestern University, UNITED STATES

## Abstract

Antibodies that recognize commensal microbial antigens may be cross reactive with a part of the human immunodeficiency virus (HIV) envelope glycoprotein gp41. To improve understanding of the role of the microbiota in modulating the immune response to HIV vaccines, we studied the associations of the gut microbiota composition of participants in the HIV Vaccine Trials Network 096 clinical trial with their HIV-specific immune responses in response to vaccination with a DNA-prime, pox virus boost strategy designed to recapitulate the only efficacious HIV-vaccine trial (RV144). We observed that both levels of IgG antibodies to gp41 at baseline and post-vaccination levels of IgG antibodies to the Con.6.gp120.B, ZM96.gp140 and gp70 B.CaseA V1-V2 antigens were associated with three co-occurring clusters of family level microbial taxa. One cluster contained several families positively associated with gp41-specific IgG and negatively associated with vaccine-matched gp120, gp140 and V1-V2-specific IgG responses. A second cluster contained families that negatively associated with gp41 and positively associated with gp120, gp140 and V1-V2-specific IgG responses. A third cluster contained microbial groups that did not correlate with any immune responses. Baseline and post-vaccination levels of gp41 IgG were not significantly correlated, suggesting that factors beyond the microbiome that contribute to immune response heterogeneity. Sequence variant richness was positively associated with gp41, p24, pg140 and V1-V2 specific IgG responses, gp41 and p24 IgA responses, and CD4+ T cell responses to HIV-1 proteins. Our findings provide preliminary evidence that the gut microbiota may be an important predictor of vaccine response.

## Introduction

The gut microbiota modulates the immune system and its response to pathogens and vaccines [1–3]. The (HIV-1) envelope glycoprotein 41 (gp41) is a target recognized by B cell epitopes during natural infection and specific GP41 antibodies are capable of neutralizing the virus, representing a natural vaccine target [4]. It has been repeatedly observed that many uninfected persons have gp41-reactive IgA and IgG before vaccination or infection [5] and emerging evidence suggests that antibodies recognizing gp41 can cross-react with commensal bacteria in the gut [6,7]. A recent HIV-1 vaccine efficacy trial of a multiclade gp140 DNA-prime and recombinant adenovirus type 5 (rAd5) boost elicited a dominant gp41-reactive antibody response that was non-neutralizing and cross-reactive with the intestinal microbiota [7]. In these studies the taxonomy of the bacteria eliciting the response were not identified as bacteria from stool were grown under anaerobic and aerobic conditions, proteins were extracted and pooled, and antibody binding to this pool was tested [7,8]. Antibodies recognizing gp41 have been shown to cross react with RNA polymerase from E. Coli, but may bind other proteins from other bacteria [6]. It remains an open question whether pre-existing gp41-reactive and or other pre-existing HIV-1-crossreactive immune responses mediated by the microbiota directly influence the vaccine-induced response. The gut microbiota has been shown to associate with vaccine responses targeting several pathogens [9–11], though not, to our knowledge, HIV.

The RV144 vaccine efficacy trial showed that an HIV-1 Env containing pox-vector prime and bivalent recombinant Env gp120 boost provided partial efficacy against HIV-1 infection [12]. Numerous follow-up studies now support the hypothesis that Env V1-V2-specific IgG were associated with decreased risk of infection among vaccine recipients [13–15]. Follow-up analyses suggest that efficacy was >60% from 3–6 months after the first vaccination, but waned over the subsequent 6–12 months [16], suggesting that improved antibody durability may improve vaccine efficacy.

The HIV Vaccine Trials Network (HVTN) 096 study [17,18] evaluated several pox prime, gp120 boost regimens with the objective of improving the immune responses elicited by the RV144 vaccine. Co-administration of AIDSVAX^®^ B/E a gp120 protein, with either a DNA plasmid or vaccinia virus containing DNA (NYVAC) encoding Env, Pol, Gag and Nef HIV-1 proteins elicited V1-V2-specific and gp120-specific IgG responses, but there was substantial heterogeneity in the response magnitude and durability among vaccine recipients. An issue with the HVTN 096 study was that the NYVAC immunogen was contaminated with mycoplasma [19], however the vaccine elicited robust Env-specific antibody responses and the study generated high quality immunogenicity data.

A critical goal of ongoing HIV-1 vaccine research is to identify factors that influence the heterogeneity in intersubject vaccine response, and the microbiota is one factor that may be involved. Vaccine induced changes in the microbiome have recently been associated with HIV/SIV (SHIV) protection in a non-human primate challenge study, and protection from SHIV infection was notably independent of the measured vaccine-induced immune responses [20]. As part of HVTN 096, the microbiota was sampled from the rectal wecks of a subset (n = 21) of the participants. Here, we examined the association between the composition and richness of the gut microbiota and the HIV-1 immune responses of these participants. We hypothesized that the levels of Env gp41-reactive antibody at baseline and the levels of vaccine-induced antibody would be associated with the bacterial composition of the gut. A principal challenge with a large multivariate dataset is to identify significant patterns without identifying spurious correlations. To mitigate against this possibility, we applied several different computational methods providing complimentary perspectives on the data, and constrained testing pre-specified classes of immune response. Using these methods, we observed correlates of vaccine protection that associated with the participants microbiota.

## Materials and methods

### Study design

HVTN 096 (ClinicalTrials.gov NCT01799954) was designed to test the safety and priming ability of either NYVAC-HIV-PT-1/NYVAC-HIV-PT-4 (hereafter NYVAC); or DNA-HIV-PT124, a trivalent bare DNA plasmid either alone or in combination with a bivalent recombinant Env gp120 protein boost (AIDSVAX^®^ B/E) [17,18]. The study contained four experimental groups each with primes administered at months 0 and 1, and boosts administered at months 3 and 6. Primes for each of the four experimental groups were (T1) NYVAC, (T2) NYVAC + AIDSVAX, (T3) DNA plasmid, (T4) DNA plasmid + AIDSVAX. In all treatments, the boost was a combination of NYVAC and AIDSVAX. An additional subset of control participants were administered placebo (Sodium chloride 0.9% solution for NYVAC placebos and 600mcg alum/mL for AIDSVAX) [17,18]. Of these treatments, T2 and T4 appeared to have the strongest immunogenicity effect [17], though immunogenicity effects of the other treatments were evident as well.

We focused on immunological measurements using samples provided at three time points: Day 0 (baseline) Month 6.5, the protocol-specified Primary Immunological endpoint (two weeks after the final boost), and Month 12 (a durability time point). While in the original experiment, there were 20 participants in each experimental group and 16 in the control group, analysis of the gut microbiota and immunological measures was limited to participants for whom both data types were available (n = 21; 7 participants in T1, 3 in T2, 6 in T3, 5 in T4; S1 Table). We analyzed all of the rectal secretion samples that were provided and available for microbiome analysis. Missing samples are assumed to be missing completely at random, thus a complete case analysis, as was performed, is unbiased. Due to the limited number of samples, data were pooled across the vaccine groups in this analysis.

### Data collection

Immune response data were generated as described by Pantaleo et al. [17]. Briefly, antibody binding was measured using the binding antibody multiplex assay (BAMA [4]). Analysis was focused on IgG and IgA levels for a subset of BAMA antigens: gp41, gag p24, CON6 gp120 B, ZM96 gp140 (encoded by the DNA and/or the NYVAC immunogen), and gp70-scaffolded clade B CaseA V1-V2 protein. Intracellular cytokine staining was performed on cryopreserved peripheral blood mononuclear cells to measure CD4+ T cell responses to vaccine-matched peptide pools for each HIV-1 protein. The proportion of cells expressing interferon gamma (IFNγ) and/or interleukin 2 (IL-2) was used as the magnitude of the response. Analysis was limited to Env-specific responses summed across three pools containing Env peptides representative of globally circulating viruses (PTE-Global[21]). Primary analysis was performed on binarized immunogenicity data, using the median to split participants into high and low categories, in order to allow for better detection of non-linear relationships between the microbiota and vaccine response. Secondary analysis was applied to Box-Cox transformed data, which was used to normalize the data and decrease sensitivity to outliers while allowing for analysis of linear trends.

DNA was extracted from wecks that were used to sample the rectal mucosa of participants to determine the gut bacterial composition (Supplement). Samples from the earliest available sample (day 0 for 3 participants, month 6.5 for 11 participants and month 12 for 7 participants; S1 Table) were amplified with barcoded primers that targeted the hypervariable sequence containing V3-V4 region of the 16S rRNA gene and sequenced on a Roche 454 [22]. Bacterial sequence data were demultiplexed, binned into sequence variants, given putative taxonomic identities, phylogenetic relationships between the sequences were ascertained and taxonomic clustering was carried out on those identities (Supplement).

### Analysis of bacterial community structure and its relationship to vaccine-induced antibody production

A two-tiered approach was used to investigate the relationship between bacterial community structure and immune responses. We employed “global” tests that identified whether overall community structure related to each immunological measurement and “local” tests, conditional on a significant global effect, to identify individual bacterial taxa underlying the effect. We focused the analysis on immunological responses that were detectable at baseline as well as post-vaccination responses that were representative of the vaccine-matched humoral or cellular response, including Env V1-V2-specific responses that previously correlated with reduced risk of infection in the RV144 HIV vaccine trial. We tested three classes of immune response:

1. Antibody binding (BAMA assay) to vaccine-matched Env gp120 (Con.6.gp120.B) and gp140 (ZM96.gp140) antigens as well as to the Env V1-V2 antigen that was a correlate of risk in the RV144 study (gp70 B. CaseA V1-V2) [13].
2. Antibody binding to two HIV-1 proteins, gp41 and p24, which, in the HVTN 096 study [17] were detectable at baseline the absence of known HIV-1 or vaccine exposure.
3. CD4+ T cell response to stimulation with pools of HIV-1 Env peptides (PTE-global). Env-specific CD4+ T cell responses were associated with reduced risk of infection in the RV144 study [23].

To describe bacterial community variability, weighted UniFrac distance was calculated between all pairs of participants. Metric multidimensional scaling (MDS), also known as principal coordinates analysis, was conducted on these weighted UniFrac distances and site scores for each axis thereof were extracted. We refer to the first ten of these weighted UniFrac MDS axes as MDS1-10 and use MDS1 in much of our subsequent analysis. Global patterns were identified with kernel regression [24] which detected community-level associations between the microbiota and immune measurements (Supplement). We also quantified each participants microbial richness and identified cases in which richness was statistically associated with each measurement, using the breakaway package [25] (Supplement).

For those immunological variables that associated with microbial community structure, local tests were applied to identify which taxa were related to each component (Supplement). Analysis of proportionality [26] was applied to identify co-occurring bacterial family level taxonomic groups, as well as to identify which of these family level groups associated with each immunologic variable found to relate to community structure in the global tests (Supplement). To adjust for multiple comparisons we computed false discovery rates FDR [27] from the *p*-values associated with each immunogenicity measurement. FDR were thus calculated separately for median split and box-cox transformed data. For the “global” tests, where we investigated whether overall community structure associated with immune variables, statistics with *p* < 0.05 and FDR < 0.2 were considered significant.

We also computed FDR for the “local” tests to control for the many species examined. At each antigen and taxonomic agglomeration level we calculated FDR from the *p*-values of the association between that antigen and each taxon of interest. With these “local” FDR calculations, our goal was to control for the many species considered, but to treat each antigen and taxonomic agglomeration level separately. While we do identify taxa with *p*<0.05 and FDR < 0.2, due to the multiple taxonomic levels that were investigated and post-hoc nature of these tests and the small sample size, we do *not* consider any specific taxa to be significantly associated and conclude rather that they are hypotheses that require validation in future studies.

## Results

### Heterogeneous immune responses before and after vaccination

**Fig 1:**
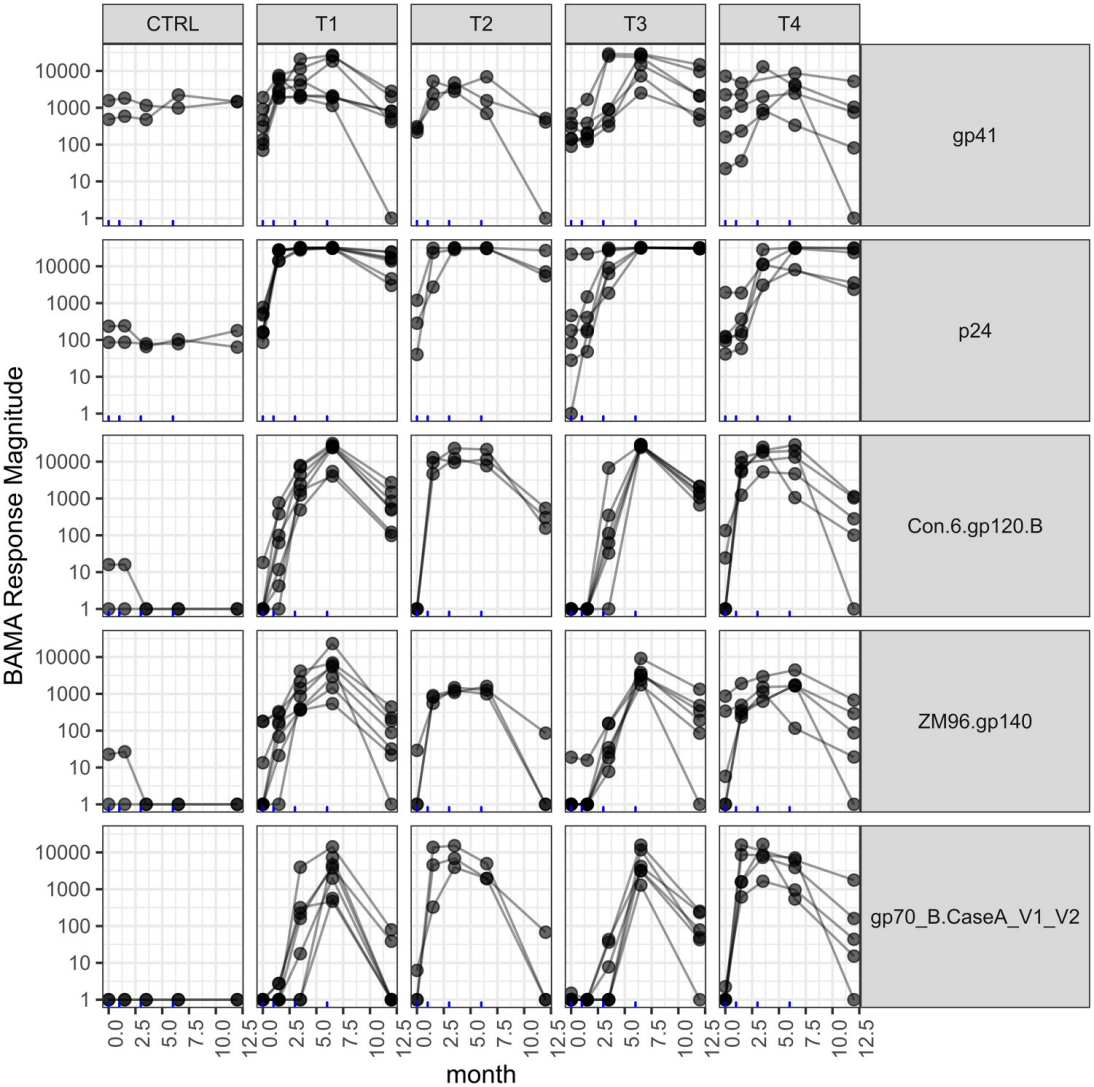
Concentration of antibodies among study participants whose gut microbiota was characterized through the BAMA assay. Participants were vaccinated at four time-points, indicated by blue tick marks along x axis, following the regimen described in the methods.

As was seen by Pantaleo et al. [17], Con 6 gp120, ZM96 V1-V2 and gp140 binding IgG antibodies were undetectable among all participants before vaccination (day 0). Relative to baseline, responses to each antigen were increased at month 6.5, two weeks after the final vaccination (Fig 1, S1A Fig), then decreased to an intermediate level at month 12. There was substantial inter-subject variability between vaccine recipients’ 6.5-month and 12-month time point antigen responses. In contrast to the other binding antibodies, gp41 and p24 binding IgG antibodies were detectable at baseline with binding increased post-vaccination. Compared to IgG, the IgA binding to gp41 and p24 varied substantially between participants at baseline increased little in response to vaccination (S1B Fig). The Env-specific CD4+ T cell response was undetectable at baseline with an increase after vaccination that attenuated by month 12, and was variable between subjects (S1B Fig). Logistic regression analysis indicated that there was no statistically detectable difference between participants that donated microbiome samples and those that did not for any immunogenicity parameter.

### Heterogeneity in gut microbiota community structure

**Fig 2:**
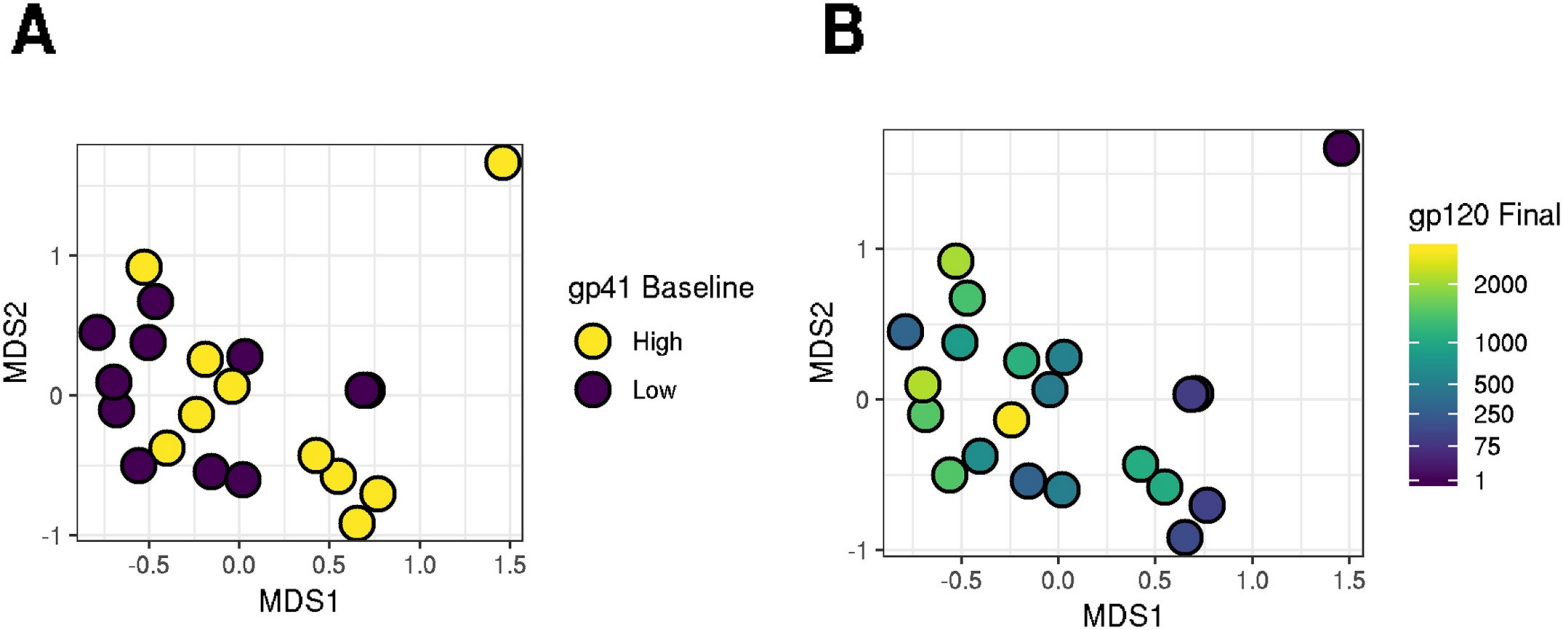
Dissimilarities between participants’ microbiota are related to dissimilarities in gp41 antibody magnitude at baseline, and gp120 magnitude at the final time-point. In this principal coordinates analysis, circles indicate participants. More distant circles indicate more dissimilar microbiota, as measured by weighted UniFrac. The first axis, MDS1 represents most of the variance, and later axes, MDS2 represents less variance. The first two axes are shown here and account for 29.1% and 17.2% of variance between participants. Positions of points in subplots are identical. Points in **A** are color coded by whether gp41 IgG concentration at day 0 is greater than or equal to (high—yellow) or lower than the median (low—blue). **B** is color coded by the Con 6 gp120 IgG concentration.

Sequencing of the V3-V4 region of the bacterial 16S rRNA gene yielded 2,100 to 10,843 reads per sample. Quality filtering, denoising and chimera removal decreased the number of reads to a final range of 2070–9688 sequences per sample. DADA2 identified 960 unique sequence variants (SVs). We removed 31 SVs that were unidentified at the Phylum level, 7 SVs from phyla that were found in the data set fewer than 20 times each (Verrumicrobia, Tenericutes, Elusimicrobia and Synergistetes), and 386 SVs that were present in fewer than 10% of the samples (as suggested by Callahan et al. [28]) yielding 536 SVs for analysis. Richness varied between participants from 151 to 441 sequence variants per sample. Confidence intervals for the richness estimates exceeded variability between participants. These sequence variants comprised 5 Phyla, 12 Classes, 17 Orders, 36 Families and 92 Genus level taxonomic groups. The participants in our dataset had microbiota broadly typical of the human gut, with most participants’ microbiota dominated by members of the Bacteroidetes and Firmicutes phyla, and some members having strong representation from the Actinobacteria and Fusobacteria phyla and the Proteobacteria Superphylum. Samples collected within the same participant at different time-points were on average more similar than samples collected from different participants (*p* = 0.02), however there was substantial variability in the weighted UniFrac distances both within and between participants (S2 Fig). Principal coordinates analysis of weighted UniFrac distances indicated that 29.1% and 16.9% of the community structure variability was captured by the first and second (MDS1 and MDS2) principal coordinate axes (Fig 2).

**Fig 3:**
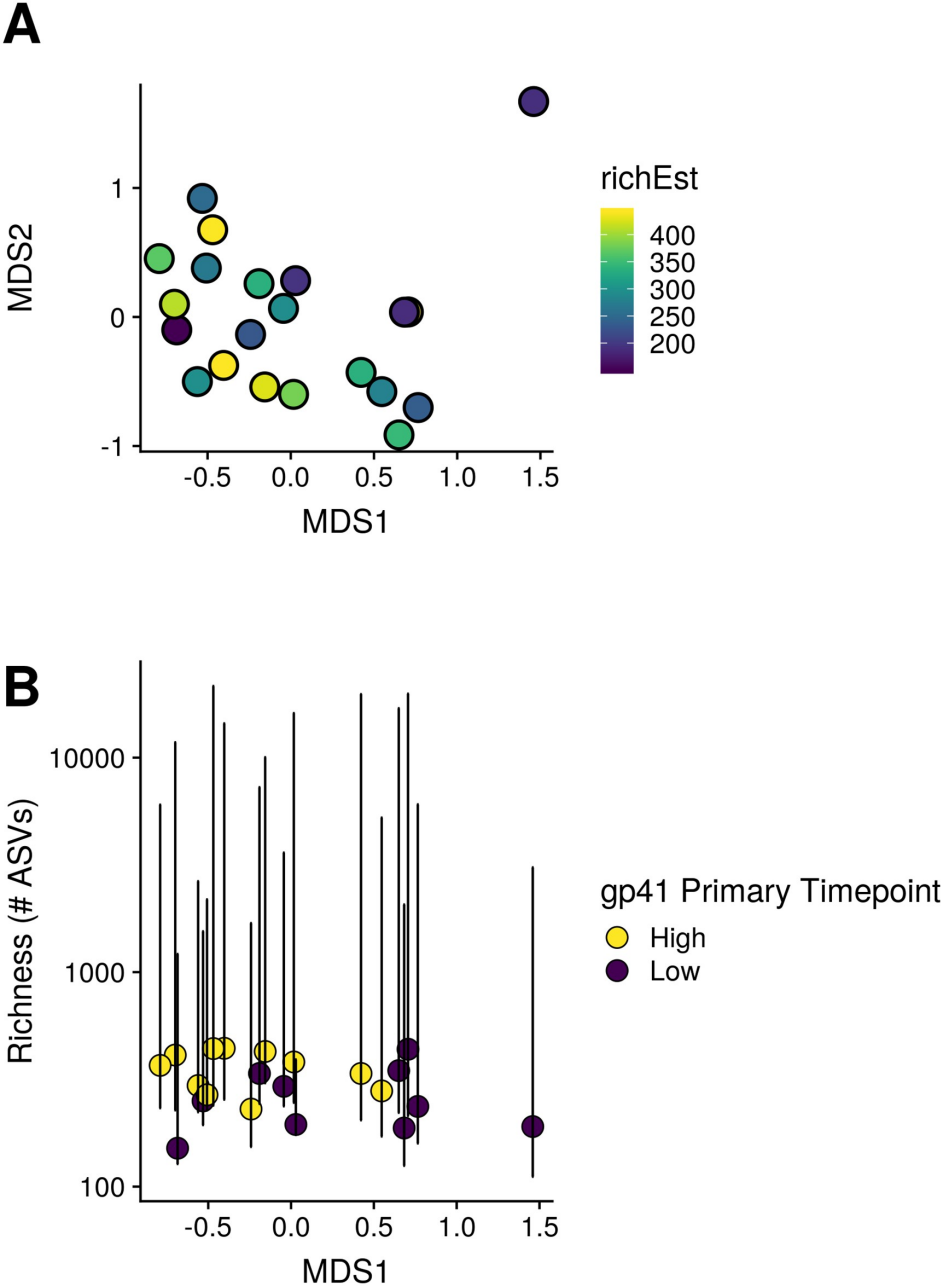
Visualization of the relationship between weighted unifrac distance, estimated richness and immunogenicity. A. The relationship between weighted unifrac distance and richness shown with principal coordinates axes. Positions of points are the same as in Fig 2, but color coding reflects species richness estimates. B. Relationship between MDS1 (x-axis), species richness, y-axis, and median split gp41 immunogenicity at the primary 6.5 month timepoint point. Error bars indicate standard errors of the richness estimates.

Sequence variant richness did not appear to associate with weighed unifrac distance, or with MDS1 (Fig 3A).

### Bacterial community structure associated with Env-reactive IgG

To identify global associations of bacterial community structure with immunological responses we used kernel regression, which tests if weighted UniFrac distances between participants are associated with the differences in their immunological responses. Each test considered “binarized” data where values lower than the median were treated as zeros and those higher than the median were treated as ones. We limited our analysis of baseline immune responses to gp41- and p24-reactive IgG and IgA since those were the only baseline responses with detectable levels. We found that baseline levels of gp41-reactive IgG were significantly associated with microbial community structure (*p* = 0.046; FDR = 0.158, Table 1, Fig 2). As a descriptive follow-up analysis, we also evaluated the association between baseline gp41 binding and MDS1, the first principal coordinate axis of community variability. We found that participants with a high MDS1 score tended to have higher gp41 binding (*p* = 0.015, FDR = 0.022; Table 1, Fig 2). Post-vaccine levels of gp41-reactive IgG at month 6.5 were also associated with community structure (*p* = 0.047, FDR = 0.158). However, compared to baseline gp41, the association of month 6.5 gp41 binding with MDS1 was in the opposite direction, with participants having high MDS1 scores tending to have lower gp41 binding (*p* = 0.032, FDR = 0.041). At the post-vaccination time points, we also assessed the associations of antibody responses to several additional HIV-1 Env antigens. We found that month 6.5 levels of Con6.gp120 and ZM96.gp140 IgG were significantly associated with community structure (gp120: *p* = 0.004, FDR = 0.073; gp140: *p* = 0.013, FDR = 0.135). IgG binding to Con6.gp120 and gp70 B.CaseA V1-V2 antigens was also associated with community structure at the month 12.5 durability time-point. For each association of a post-vaccination Env-reactive IgG we found that participants with higher MDS1 scores tended to have lower Env-specific IgG while those with lower MDS1 scores had higher Env-specific IgG. We found no association between the Env-specific CD4+ T cell response or any IgA measurements with community structure.

**Table 1:** Significance values of kernel regression tests, and McFadden’s R2 values, slope coefficients and significance values of logistic regression models show that magnitude of response for several IgG antibodies solicited by vaccines appear to relate to the structure of the microbiota. In these tests, antibody levels were treated as binary, median split variables. Kernel regression asked whether participants that both have high or low concentrations of the antibody or T cell pool of interest have similar microbiota (as measured by weighted UniFrac) than participants with dissimilar concentrations of that variable of interest. Logistic regression GLMs ask whether the weighted UniFrac axis 1 (MDS1) scores (Fig 1) of the participants are statistically related the variables of interest. The logistic regression GLM coefficients tell us of the direction and strength of the association. R22 indicates Nagelkerke pseudo R2, p-values are calculated by permutation (Kernel regression) and directly (weighted Unifrac Regression), and Benjamini and Holchberg FDR values [27] are calculated using the bioconductor q-value package [29]. Bold text and yellow shading indicate statistically significant p (<0.05) and FDR (<0.20) values. Regression coefficients corresponding kernel p < 0.05 and FDR < 0.2, values are color-coded according to their sign; blue = positive, red = negative.

			Kernel		MDS			
Type	Antigen	Month	P	FDR	P	FDR	R^2^	Coef
CD4+	Any ENV PTEG	6.5	0.251	0.493	0.218	**0.125**	0.093	-0.937
		12	0.246	0.493	0.228	**0.125**	0.094	-0.920
IgA	gp41	0	0.957	0.957	0.651	0.219	0.013	0.333
		6.5	0.207	0.493	0.143	**0.109**	0.129	-1.133
		12	0.746	0.933	0.855	0.259	0.002	-0.136
	p24	0	0.895	0.951	0.320	**0.161**	0.061	0.752
		6.5	0.903	0.951	0.650	0.219	0.013	-0.333
		12	0.386	0.593	0.387	**0.172**	0.049	-0.657
IgG	Con.6.gp120.B	6.5	**0.004**	**0.078**	**0.002**	**0.011**	0.497	***-3*.*104***
		12	**0.036**	**0.157**	**0.010**	**0.017**	0.361	***-2*.*289***
	gp41	0	**0.047**	**0.157**	**0.014**	**0.017**	0.331	**2.185**
		6.5	**0.047**	**0.157**	**0.030**	**0.031**	0.267	***-1*.*809***
		12	0.648	0.864	0.779	0.248	0.005	-0.205
	gp70 B.CaseA V1-V2	6.5	0.904	0.951	0.619	0.219	0.016	-0.365
		12	**0.035**	**0.157**	**0.014**	**0.017**	0.332	***-2*.*135***
	p24	0	0.215	0.493	0.444	**0.179**	0.037	-0.567
		6.5	0.418	0.597	0.397	**0.172**	0.047	-0.749
		12	0.286	0.493	0.159	**0.109**	0.120	-1.085
	ZM96.gp140	6.5	**0.017**	**0.157**	**0.009**	**0.017**	0.370	***-2*.*338***
		12	0.296	0.493	0.162	**0.109**	0.118	-1.076

Treating immune data as a continuous, box-cox transformed, rather than binarized variables showed similar though generally weaker correlation patterns. One notable exception was IgG antibodies to Con.6.gp120B which showed a much stronger association (*p*<0.001, FDR < 0.01) when treated as a continuous, rather than binary variable (S2 Table).

**Fig 4:**
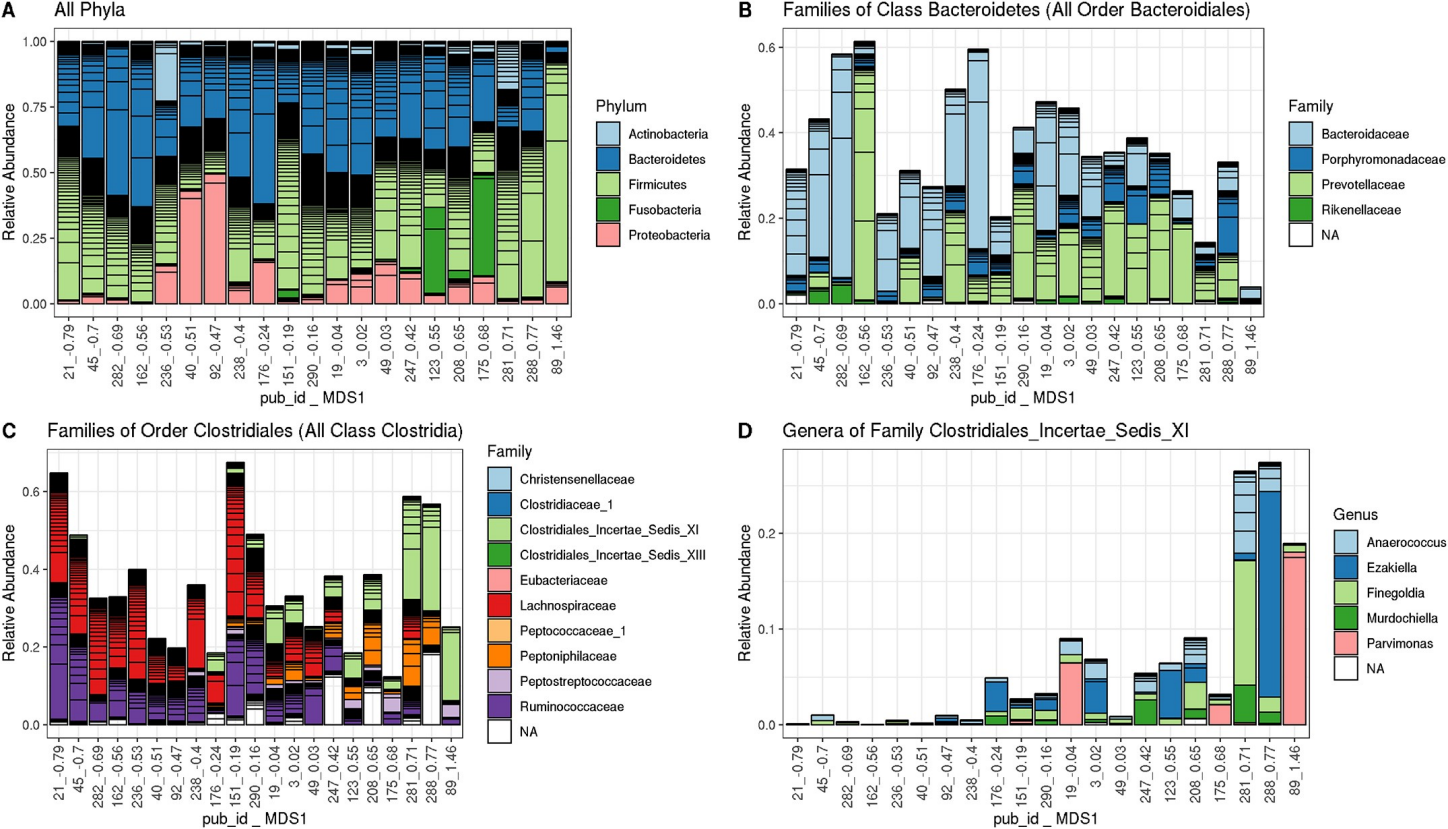
The microbiota vary between participants and can be described by weighted UniFrac axis 1 (MDS1). Variations along this axis are evident at the family level within certain classes and orders, but are less evident at the phylum or genus level. These stacked bar plots show relative abundance of taxonomic groups composing participants’ microbiota. In all cases participants are ordered from left to right according to where their microbiota falls along weighted MDS1 (Fig 2). A All taxa at phylum level. B. All families within Order Clostridiales, all of which fall within Class Clostridia. C. All families within Order Bactereoidales, all of which are in the Class Bacteroidetes. D. Genera within the family Clostridiales Incertae Sedis XI.

We used samples’ locations along MDS1 to aid visualization of community structure (Fig 4). Participants with low MDS1 scores had Clostridiales communities dominated by the Ruminococcaceae and Peptoniphilaceae families, while participants with high MDS1 scores had higher levels of Clostridiales Incertae Sedis XI (Fig 4B). Surprisingly, while variability along MDS1 was evident at the family level, this variability did not extend to the genera within families. For instance, the Clostridiales Incertae Sedis XI were primarily composed of six genera and the relative contributions of these genera to Clostridiales Incertae Sedis XI were not associated with MDS1 score (Fig 4C).

Of the Bacteroidetes seen in this dataset, most were from Class Bacteroidia and all of those Bacteroidia were from order Bacteroidales. The remainder of Bacteroidetes were from a small subset of otherwise unidentified organisms. Within these Bacteroidetes, variability was also evident along MDS1. When sorted by weighted MDS1 score, it was evident that participants with low MDS1 scores generally had higher levels of the Family Bacteroidaceae, while participants with higher MDS1 scores tended to have higher concentrations of the family Prevotellaceae (Fig 4D).

As a secondary analysis we used kernel regression to assess the association of the immune responses with community structure using a Jensen-Shannon distance, which does not incorporate phylogenetic relationships among SVs. We noted that this analysis did not identify any associations, however when we repeated the analysis, agglomerating SVs at the phylum, class, order, family and genus levels some notable correlations emerged, with the strongest correlations at the family level. Omnibus *p*-values providing a summary *p*-value across taxonomic levels generally provided *p*-values that were close to, but slightly higher than, the results of the weighted UniFrac based kernel regression (S4 Fig).

We observed a positive relationship between the richness of sequence variants and the magnitude of immunogenicity responses (Table 2, S4 Table, Fig 3B).

**Table 2:** Coefficients, R2 values and significance values of the relationship between alpha diversity and median-split immunogenicity responses calculated using the betta function in breakaway. Highlighting and FDR calculations were applied as described in Table 1. R2 values were calculated from the pearson correlation coefficient between alpha diversity and median split immunogenicity values.

Type	Antigen	Month	Coef	R2	P	FDR
CD4+	Any ENV PTEG	6.5	**40.69**	0.033	**0.041**	**0.091**
		12.0	**44.76**	0.006	**0.013**	**0.037**
IgA	gp41	0.0	-1.84	0.032	0.946	0.946
		6.5	16.30	0.145	0.534	0.628
		12.0	**68.38**	0.211	**0.017**	**0.042**
	p24	0.0	5.46	0.007	0.827	0.871
		6.5	**56.58**	0.16	**0.010**	**0.033**
		12.0	32.85	0.014	0.168	0.280
IgG	Con.6.gp120.B	6.5	21.04	0.003	0.348	0.535
		12.0	17.55	0.001	0.440	0.587
	gp41	0.0	17.12	0.001	0.503	0.628
		6.5	**76.38**	0.247	**0.001**	**0.010**
		12.0	**61.02**	0.094	**0.009**	**0.033**
	gp70 B.CaseA V1-V2	6.5	-5.83	0.006	0.812	0.871
		12.0	**70.51**	0.062	**0.000**	**0.000**
	p24	0.0	-30.85	0.033	0.129	0.235
		6.5	**60.60**	0.117	**0.010**	**0.033**
		12.0	47.26	0.234	0.074	**0.148**
	ZM96.gp140	6.5	21.89	0.036	0.387	0.553
		12.0	**66.78**	0.095	**0.002**	**0.013**

### Individual taxonomic groups were associated with gp41 and gp120-reactive IgG binding

We assessed the association of each species-level SV with each of the IgG responses that were found to significantly covary with the community. No species-level SVs were associated with an IgG immune response after multiplicity adjustment. In contrast, with taxonomic agglomeration, it was evident that each IgG variable was associated with multiple taxa at each of several taxonomic levels (S5 Fig). All IgG antigen-time point combinations that were statistically associated with the overall community, according to global tests, were also associated with some family level taxa (*p* < 0.05, FDR < 0.2). Thus, subsequent analyses, were focused on family level patterns.

**Fig 5:**
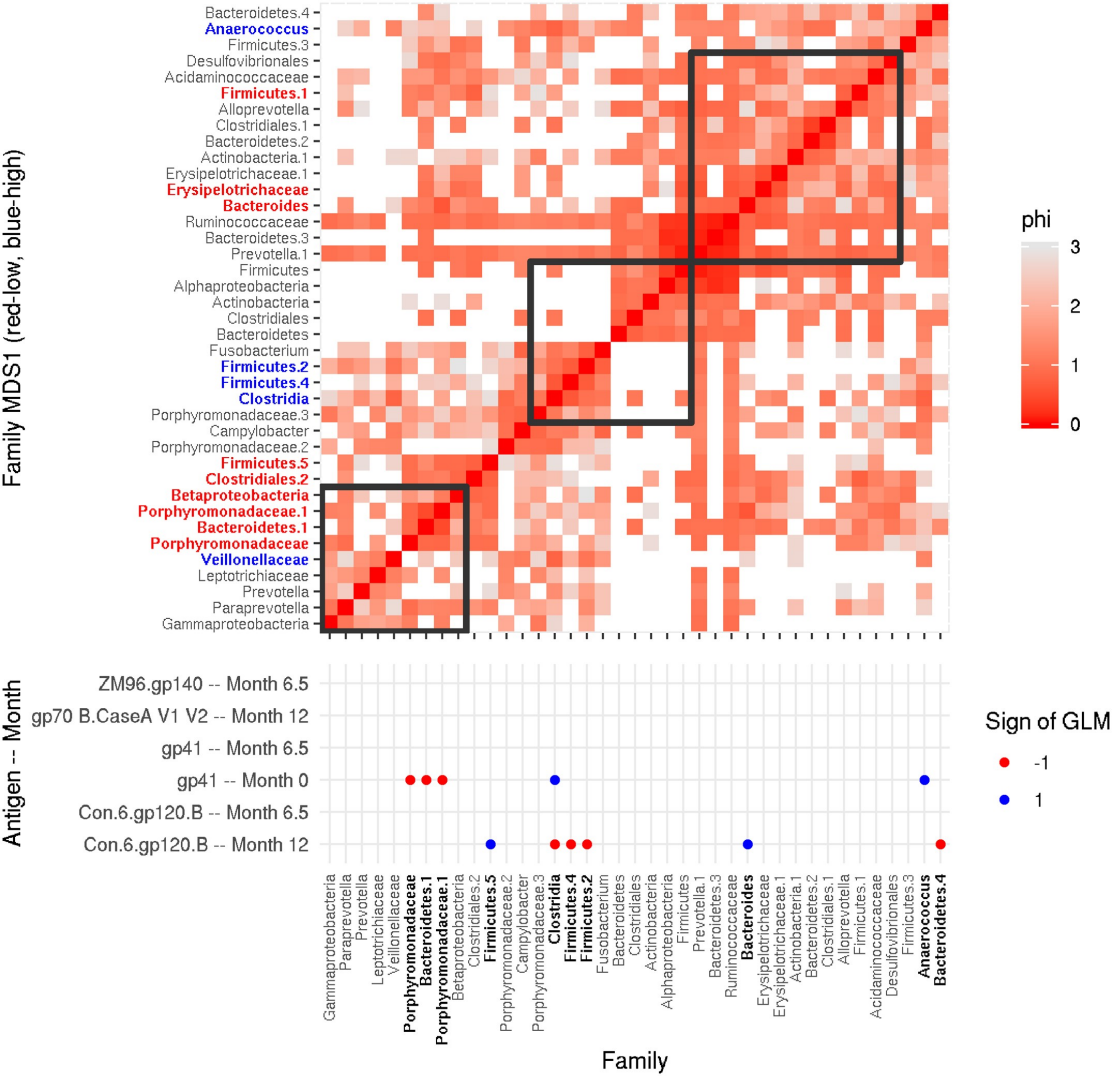
Family level groups fall into three proportional clusters, two of which are associated, in opposite ways, with magnitude of IgG responses as demonstrated by a Heat-map of Φ [26], a measure of proportionality between different family level groups. In the upper panel (heatmap), each row and column are a family level taxon. Redder cells indicate lower Φ scores, which demonstrate higher statistical association between the pair of families. Three proportional clusters (surrounded by squares) can be seen. The dots in the lower panel (dot-grid) indicate IgG binding antibodies specific to each antigen at each time points (y-axis) that associate with each family level taxon (x-axis) (*p* < 0.05, FDR < 0.2). Blue dots indicate that a family level group is positively associated with a particular antibody response at a given time point, while red dots indicate negative associations. Slope coefficients of these logistic associations are reported in S6 Fig. Bacterial families in the top right cluster associate positively with magnitude of response to the vaccine target antigens Con.6.Gp120.B, gp70 B.Case A V1-V2, and ZM96.gp140 and gp41 at month 6.1 and negatively to gp41 at baseline. The middle cluster associate negatively with IgG response to vaccine target antigens and positively to gp41 baseline response. The lower left cluster does not appear to associate with any of the antigens.

We used an analysis of proportionality to better understand the community structure. In this analysis, we determined which family level groups were positively associated with each other according to a “proportionality” metric that is robust to compositional effects [26]. At the family level there were three main co-occurring clusters in the data (Fig 5). One cluster contained families that were positively associated with gp41 day 0 responses. This same cluster also contained family level groups that were negatively associated with gp41 at the 6.5-month time point, and groups that were positively associated with Con.6.gp120.B, ZM96.gp140 and gp70 B.CaseA V1-V2 levels. In contrast, families in the other clusters were negatively associated with gp41 and positively associated with the other immune variables. A third cluster appeared to be independent of the measured immune responses.

## Discussion

The statistical association between the microbiome and gp41 specific IgG levels at the baseline visit in our study supports previous indications that the microbiome shapes immune system development and function (e.g. [30]). To our knowledge, this paper is the first to identify a correlation between baseline gp41 binding IgG antibodies and the microbiome. The observed association between the richness and community structure of the microbiota and immunogenicity of the NYVAC containing vaccine regimens expands on previous studies suggesting that the microbiome influences the immune system and is associated with vaccine immunogenicity.

Both phylogenetically informed and phylogenetically agnostic methods are standard in the analysis of microbial beta diversity [31,32], and have been shown to provide complementary information in some systems [33]. Our analysis of the relationship between taxonomic agglomeration and strength of associations between immunogenicity and microbiome (Section 2.4). demonstrated that without a phylogenetic informed distance metric like UniFrac, taxonomic agglomeration was necessary to see patterns. That is, we detected no relationship between species level SVs and immunologic parameters when we ignored their phylogenetic relationships. In contrast, when we accounted for phylogeny with methods based on weighted UniFrac [34] or phylogenetic agglomeration of SVs, relationships between community structure and vaccine response were evident.

Groups of co-occurring organisms were observed both as proportional clusters of family level taxa (Fig 4) and as metric MDS axes in weighted UniFrac space (Fig 2). These groups were associated with vaccine response, as measured by the production of a variety of antigen-specific antibodies, and were inversely correlated with baseline gp41 antibody levels. While our statistical analyses identified just a few organisms within these groups as relating to immunogenicity, given the small sample size it is possible that other members of the cluster are also associated with the immune response.

The baseline and vaccine-induced IgG response were inversely associated with the microbiota (Table 1, Fig 4). Within the family level cluster analysis, groups of families that were associated with high concentrations of gp41-reactive IgG were also associated with low levels of gp120-reactive IgG at the 6.5 and 12-month time points. Surprisingly, bacteria associated with high baseline gp41 IgG were associated with low month 6.5 gp41 IgG. Despite these opposite correlations with the microbiota, direct assessment of the baseline and post-vaccine correlation showed that levels of baseline gp41-reactive IgG were not predictive of any of the post-vaccine IgG responses. Such a non-equality is possible because the correlations are weak and not all of the variability can be explained by the microbiota measurements.

With 21 individuals providing both immune response and microbiota data, the study had limited power. Furthermore, the small sample size limited detection of “local” level patterns and forced us to make broader comparisons by looking at proportional family level groups and weighted UniFrac measurements that summarize “overall” community structure rather than specific organisms. Future studies with larger sample sizes will be needed to identify the specific bacterial taxa underlying the patterns we observed.

The false discovery rate controls for the number of false positives that fall below that cutoff, and indeed up to 20% of our global observations may have been false positives. While some of our observations may be spurious, our broad findings, with the above caveats about samples size, indicate that immunogenicity variables do associate with microbiota.

Our study used amplicon analysis, which is subject to primer bias which can lead to variability in the observed presence and abundance of some sequence variants. Indeed, we recognize that whole genome shotgun sequencing can provide much of the information generated here with less bias, and that it may also provide additional information about microbial traits. Indeed, we advocate using newer technologies in subsequent analysis. At the time the initial analysis was performed as well as in the present day, however amplicon analysis provides substantial cost savings. Furthermore, we were focused on looking at whether differences in the microbiota appeared to associate with immunogenicity, and we expect that this core finding would hold regardless of sequencing method.

A key limitation of the HVTN 096 study overall was that, near completion of the clinical trial, the NYVAC vaccine was discovered to be contaminated with mycoplasma [17,19]. This contamination could modulate the interaction between the immune system and microbiota in this study in a way that it might not with vaccines in the future.

While the microbiome has been shown to impact efficacy of oral vaccines [9,10,35] this is the first study, to our knowledge, to demonstrate statistical association between microbiota structure and immunogenicity of an HIV vaccine, as well as the first to demonstrate the effect of the microbiota on a vaccine delivered parenterally. Future vaccine development studies, especially HIV-1 vaccine studies, should consider the microbiome as a potential correlate of immunogenicity or correlate of protection.

## Supporting information

S1 Fig Magnitude of vaccine responses over time for all study participants. **A** Concentration of IgG binding antibodies over time and **B** concentration of IgA binding antibodies, and Env specific CD4+ Helper cells among all study participants, including those without microbiota sequencing.
(PDF)

S2 Fig Variability in microbiota within and between participants. Weighted unifrac distances between samples taken within the same participant at different time points (“within”), and between different participants from all time points (“between)—open circles. Black squares indicate the mean unifrac distance for the “within” and “between” participants samples. Violins show the bootstrapped distributions of those means. Samples taken between participants have a weighted UniFrac distance that is 0.087 larger than those taken from within the same participant (bootstrapped 95% confidence interval: 0.012–0.154; permutation based *p* = 0.02).
(PDF)

S3 Fig Q-Q Plots of kernel regression *p*-values. Q-Q Plots comparing observed to expected kernel regression *p*-values for both (A) logistic (Corresponding to Table 1) and (B) gaussian glm models (corresponding to S2 Table). The diagonal is the 1:1 line. Points below the diagonal indicate associations with *p*-values that were lower than expected from a uniform distribution of *p*-values.
(PDF)

S4 Fig Relationship between taxonomic aggregation and statistical significance of microbiota-vaccine associations. Kernel regression *p*-values of using kernels calculated from Jensen-Shannon distance matrices calculated from SV tables that have been agglomerated to different (Phylum through Species level) taxonomic levels. Squares indicate omnibus *p*-values, which indicate whether there is a statistically meaningful hit at any taxonomic level, adjusting for multiple comparisons [2]. Triangles indicate kernel regression *p*-values for weighted UniFrac scores, and are identical to ones reported in Table 1 and S1 Table.
(PDF)

S5 Fig Relationship between taxonomic aggregation and statistical significance of individual microbe—Vaccine associaitons. Statistical significance of regressions of taxa agglomerated at a range of taxonomic levels, against each antibody found to relate to community structure in Table 1 and S1 Table. We report both **A** *p*-values and **B** False discovery rates for each. False discovery rates are calculated from *p*-values at each antibody-taxonomic level combination. Horizontal lines indicate significance thresholds of 0.2 (gray), 0.05 (blue) and 0.01 (green).
(PDF)

S6 Fig Coefficients of statistically significant, family level, microbe-vaccine associations. Coefficients (y-axis) of general linear models relating family level taxa (x-axis) to antibody concentrations for which there was at least one statistically significant hit in S5 Fig. Error bars represent two standard errors of the coefficient. Colors and shapes indicate whether models have *p*-values < 0.05 and FDR < 0.2, respectively. Only taxa involved in at least one statistically significant association are shown. These same families are indicated with dots in Fig 4 when they are statistically significant.
(PDF)

S1 Table Stool donors by time and treatment. Numbers of participants per vaccine treatment group (columns), and time points from which their microbiota were collected for this study (rows). Treatment codes, T1-T4, are described in Section 1.1.
(PDF)

S2 Table Gaussian linear model results. Significance values of kernel regression tests, as well as significance values and slope coefficients of gaussian general linear models. This table mirrors Table 1, with the exception that here, antibody concentrations are treated as continuous, box-cox transformed variables, rather than binomial, median split variables. Kernel regressions ask whether participants that both have high or low concentrations of the antibody or T cell pool of interest have more similar microbiota (as measured by weighted UniFrac) than participants with dissimilar concentrations of that variable of interest. GLMs ask whether the weighted UniFrac axis 1 (MDS1) scores (Fig 1) of the participants are statistically related the variables of interest. Coefficients show the strength and direction of the association. R^2^ indicates Nagelkerke pseudo R^2^. *p*-values are calculated by permutation (Kernel regression) and directly (weighted Unifrac Regression). BH-FDR values are calculated from the *p*-values using the bioconductor q-value package. Yellow highlighting indicates statistically significant *p* (<0.05) and FDR (<0.20) values. Regression coefficients corresponding kernel *p* < 0.05 and FDR *< 0*.*2*, values are color-coded according to their sign, red = negative, there are no positive coefficients meeting our significance thresholds.
(PDF)

S3 Table Only MDS1 of the microbiota predicts immunogenicity. *p*-values of logistic regressions between UniFrac PCoA components 1–10 (MDS1-MDS10) and the median split transformed concentrations of the antibodies described in Table 1. MDS1 is associated with several immune responses (*p* < 0.05, FDR < 0.2), the other MDS components are not.
(PDF)

S4 Table Microbial alpha diversity’s relationship to continuous, rather than median split discrete, immunogenicity variables. Coefficients, R^2^ values and significance values of the relationship between alpha diversity and box-cox transformed and then z-normalized immunogenicity responses calculated using the betta function in breakaway. Highlighting and FDR calculations were applied as described in Table 1. R^2^ values were calculated from the pearson correlation coefficient between alpha diversity and median split immunogenicity values.
(PDF)

S1 Methods Supplemental methods section. (PDF)
