# Supplementary material for: Human gut microbiota is associated with HIV-reactive immunoglobulin at baseline and following HIV vaccination
Source: PLoS One. 2019 Dec 23;14(12):e0225622. doi: 10.1371/journal.pone.0225622 (PMC6927600; doi:10.1371/journal.pone.0225622)
Supplement: S1 Fig — A Concentration of IgG binding antibodies over time and B concentration of IgA binding antibodies, and Env specific CD4+ Helper cells among all study participants, including those without microbiota sequencing. (PDF) [file pone.0225622.s001.pdf]

A

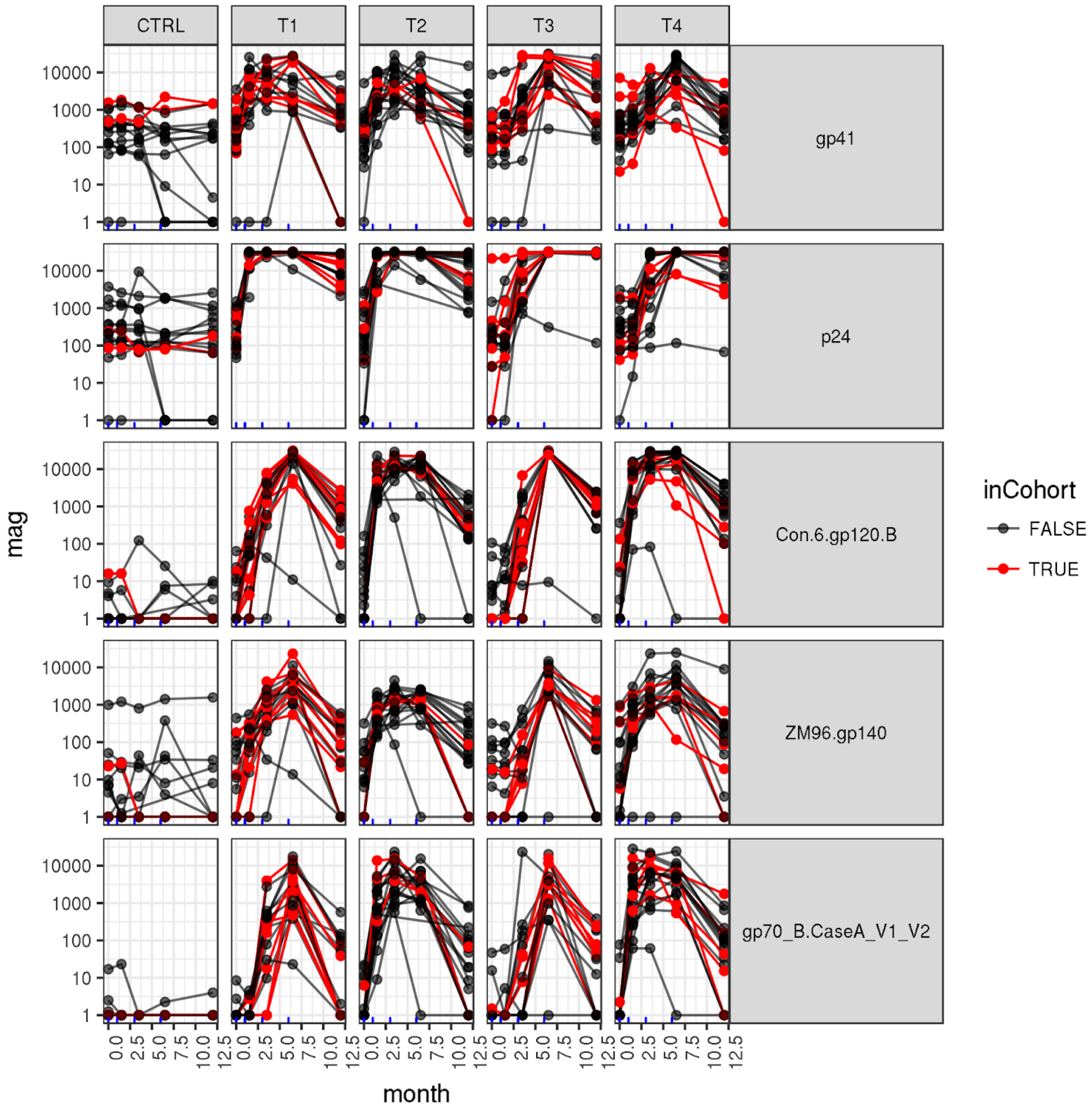
CTRL
T1
T2
T3
T4
gp41
p24
Con.6.gp120.B
ZM96.gp140
gp70_B.CaseA_V1_V2
mag
10000
1000
100
10
1
month
0.0
2.5
5.0
7.5
10.0
12.5
inCohort
FALSE
TRUE

B

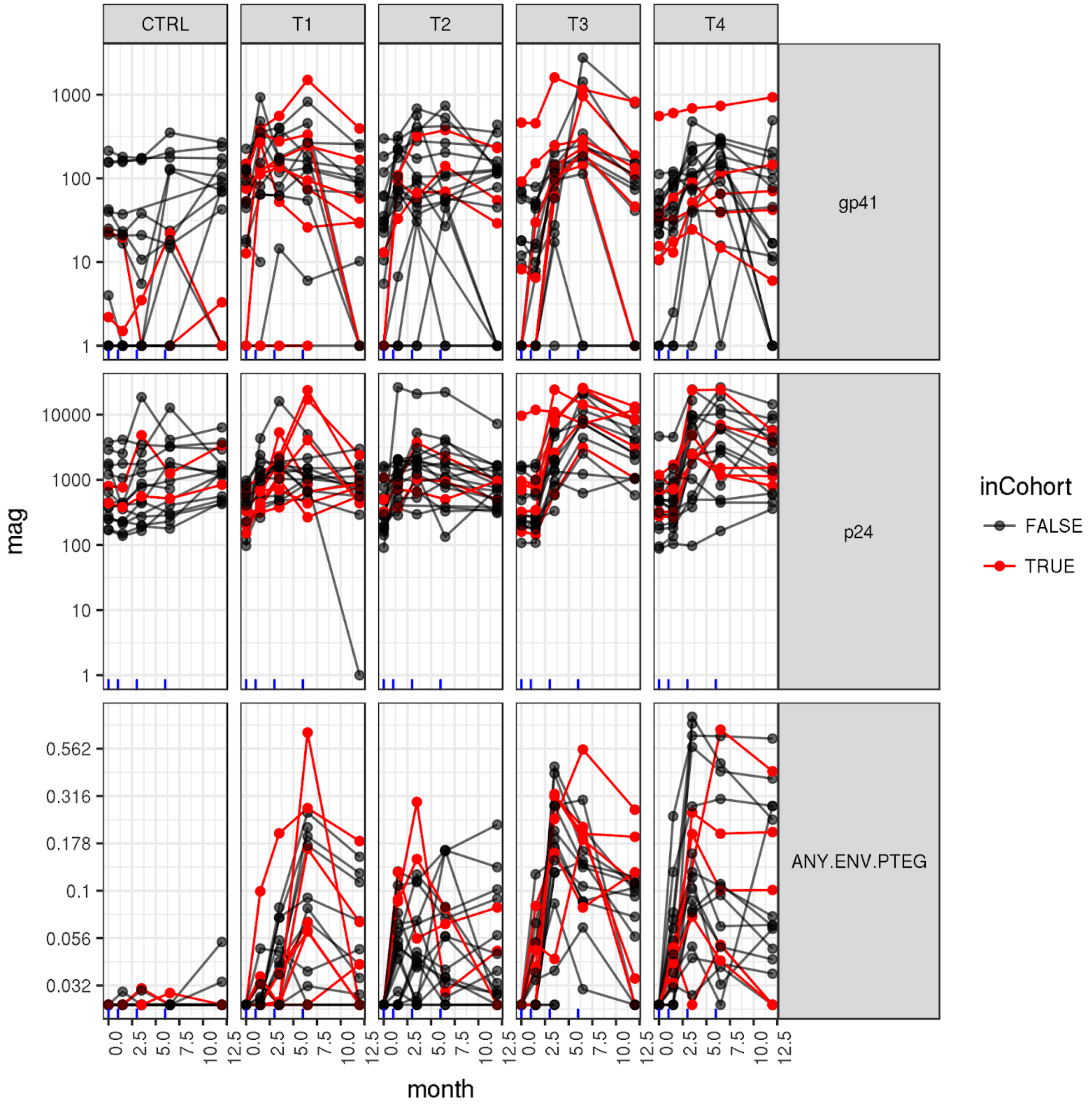

S1 Fig. **A** Concentration of IgG binding antibodies over time and **B** concentration of IgA binding antibodies, and Env specific CD4+ Helper cells among all study participants, including those without microbiota sequencing.
