# Supplementary material for: Human gut microbiota is associated with HIV-reactive immunoglobulin at baseline and following HIV vaccination
Source: PLoS One. 2019 Dec 23;14(12):e0225622. doi: 10.1371/journal.pone.0225622 (PMC6927600; doi:10.1371/journal.pone.0225622)
Supplement: S2 Fig — Weighted unifrac distances between samples taken within the same participant at different time points (“within”), and between different participants from all time points (“between)—open circles. Black squares indicate the mean unifrac distance for the “within” and “between” participants samples. Violins show the bootstrapped distributions of those means. Samples taken between participants have a weighted UniFrac distance that is 0.087 larger than those taken from within the same participant (bootstrapped 95% confidence interval: 0.012–0.154; permutation based p = 0.02). (PDF) [file pone.0225622.s002.pdf]

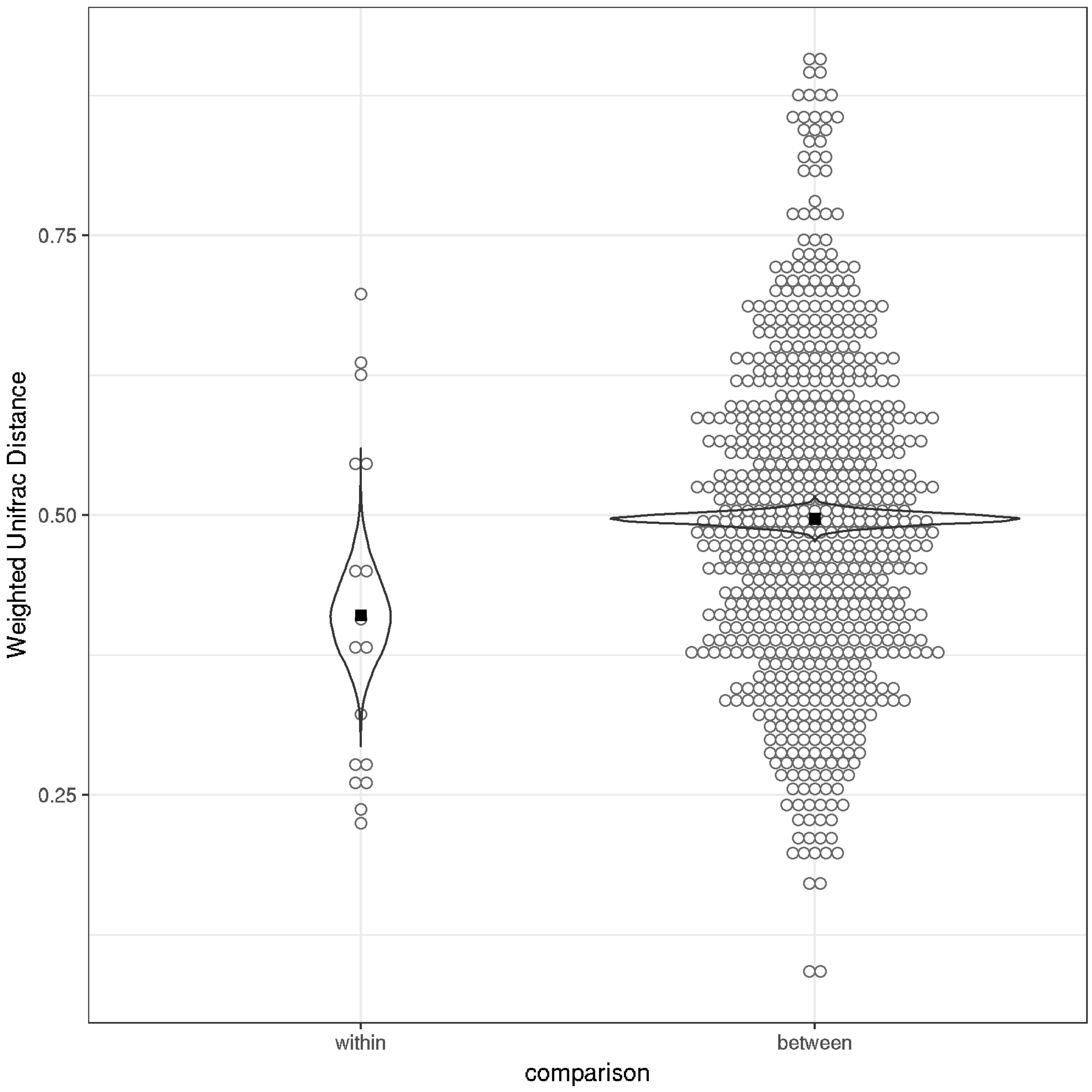

S2 Fig. Weighted unifrac distances between samples taken within the same participant at different time points (“within”), and between different participants from all time points (“between) -- open circles. Black squares indicate the mean unifrac distance for the “within” and “between” participants samples. Violins show the bootstrapped distributions of those means. Samples taken between participants have a weighted UniFrac distance that is 0.087 larger than those taken from within the same participant (bootstrapped 95% confidence interval: 0.012-0.154; permutation based $p = 0.02$).
