# Supplementary material for: Human gut microbiota is associated with HIV-reactive immunoglobulin at baseline and following HIV vaccination
Source: PLoS One. 2019 Dec 23;14(12):e0225622. doi: 10.1371/journal.pone.0225622 (PMC6927600; doi:10.1371/journal.pone.0225622)
Supplement: S3 Fig — Q-Q Plots comparing observed to expected kernel regression p-values for both (A) logistic (Corresponding to Table 1) and (B) gaussian glm models (corresponding to S2 Table). The diagonal is the 1:1 line. Points below the diagonal indicate associations with p-values that were lower than expected from a uniform distribution of p-values. (PDF) [file pone.0225622.s003.pdf]

A

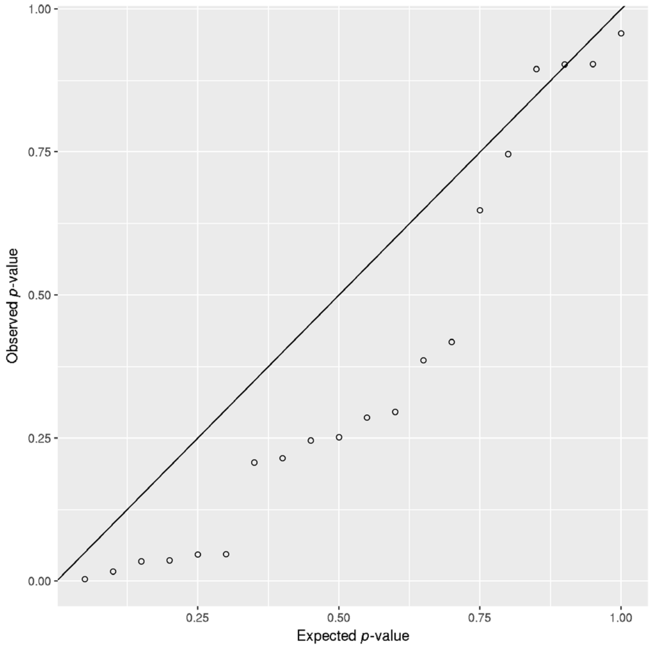

B

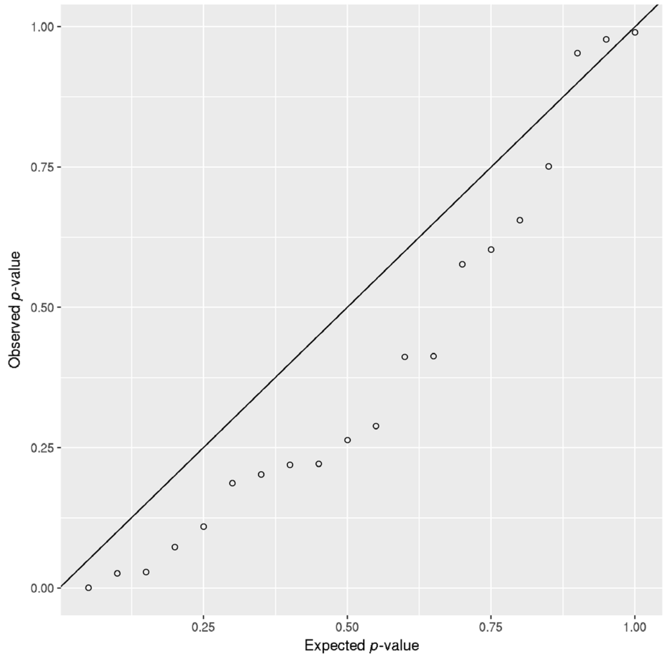

S3 Fig. Q-Q Plots comparing observed to expected kernel regression *p*-values for both (A) logistic (Corresponding to Table 1) and (B) gaussian glm models (corresponding to Table S2). The diagonal is the 1:1 line. Points below the diagonal indicate associations with *p*-values that were lower than expected from a uniform distribution of *p*-values.
