# Supplementary material for: Human gut microbiota is associated with HIV-reactive immunoglobulin at baseline and following HIV vaccination
Source: PLoS One. 2019 Dec 23;14(12):e0225622. doi: 10.1371/journal.pone.0225622 (PMC6927600; doi:10.1371/journal.pone.0225622)
Supplement: S4 Fig — Kernel regression p-values of using kernels calculated from Jensen-Shannon distance matrices calculated from SV tables that have been agglomerated to different (Phylum through Species level) taxonomic levels. Squares indicate omnibus p-values, which indicate whether there is a statistically meaningful hit at any taxonomic level, adjusting for multiple comparisons [2]. Triangles indicate kernel regression p-values for weighted UniFrac scores, and are identical to ones reported in Table 1 and S1 Table. (PDF) [file pone.0225622.s004.pdf]

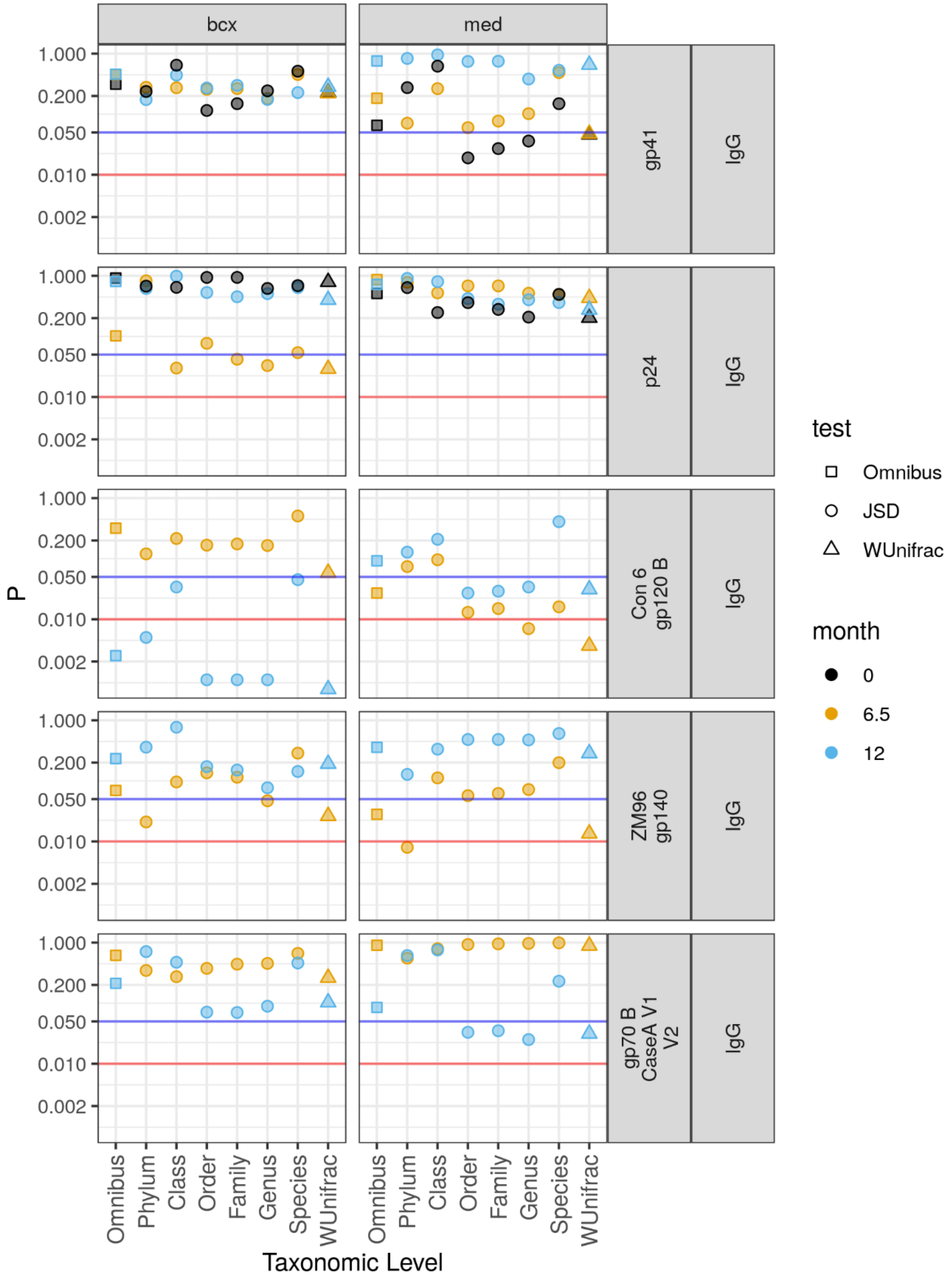

S4 Fig. Kernel regression *p*-values of using kernels calculated from Jensen-Shannon distance matrices calculated from SV tables that have been agglomerated to different (Phylum through Species level)

taxonomic levels. Squares indicate omnibus *p*-values, which indicate whether there is a statistically meaningful hit at any taxonomic level, adjusting for multiple comparisons [2]. Triangles indicate kernel regression *p*-values for weighted UniFrac scores and are identical to ones reported in Tables 1 and S1.
