# Supplementary material for: Human gut microbiota is associated with HIV-reactive immunoglobulin at baseline and following HIV vaccination
Source: PLoS One. 2019 Dec 23;14(12):e0225622. doi: 10.1371/journal.pone.0225622 (PMC6927600; doi:10.1371/journal.pone.0225622)
Supplement: S5 Fig — Statistical significance of regressions of taxa agglomerated at a range of taxonomic levels, against each antibody found to relate to community structure in Table 1 and S1 Table. We report both A p-values and B False discovery rates for each. False discovery rates are calculated from p-values at each antibody-taxonomic level combination. Horizontal lines indicate significance thresholds of 0.2 (gray), 0.05 (blue) and 0.01 (green). (PDF) [file pone.0225622.s005.pdf]

A

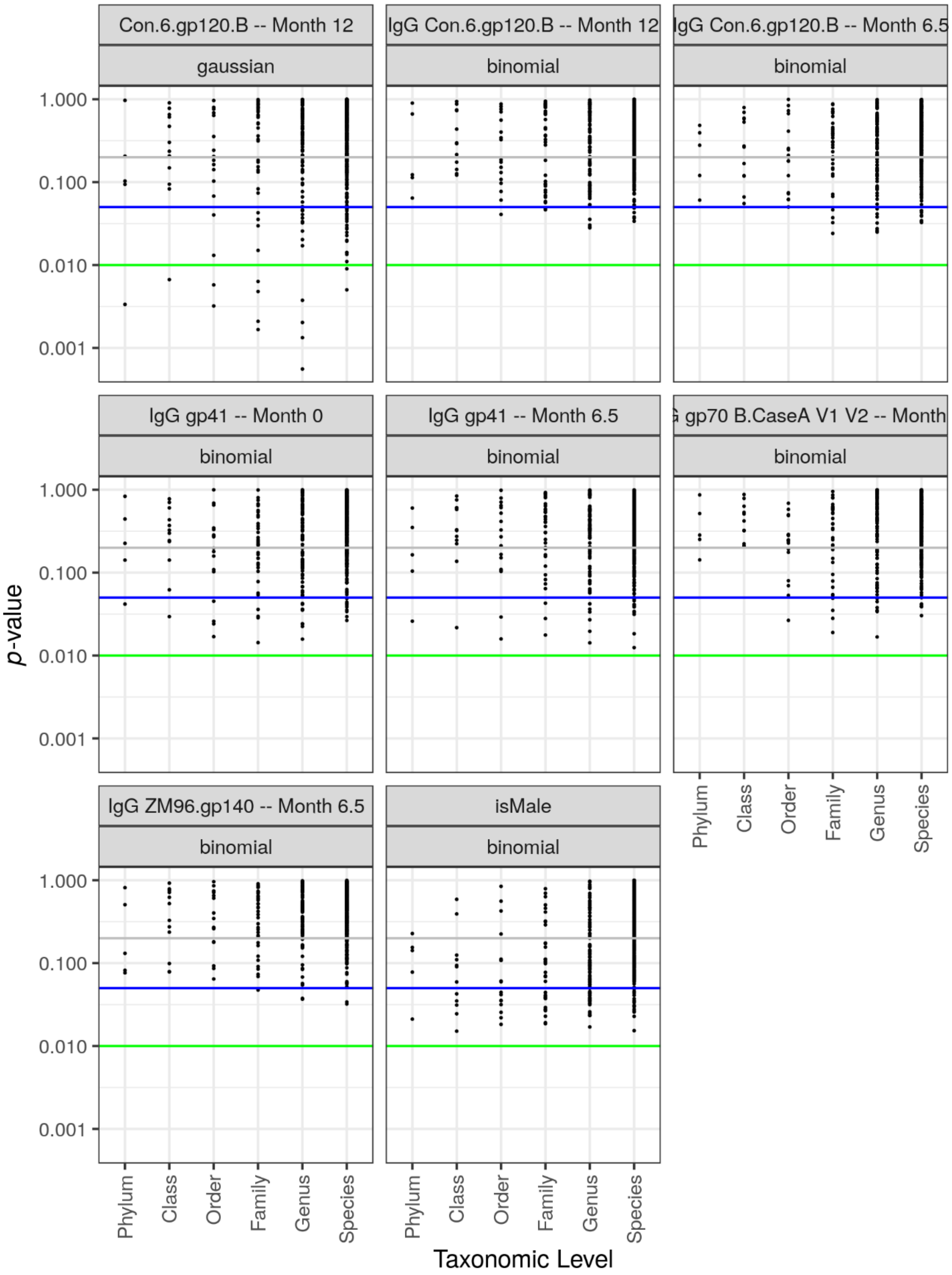

Con.6.gp120.B -- Month 12
gaussian
IgG Con.6.gp120.B -- Month 12
binomial
IgG Con.6.gp120.B -- Month 6.5
binomial
IgG gp41 -- Month 0
binomial
IgG gp41 -- Month 6.5
binomial
gp70 B.CaseA V1 V2 -- Month
binomial
IgG ZM96.gp140 -- Month 6.5
binomial
isMale
binomial
1.000
0.100
0.010
0.001
p-value
Phylum
Class
Order
Family
Genus
Species
Taxonomic Level

B

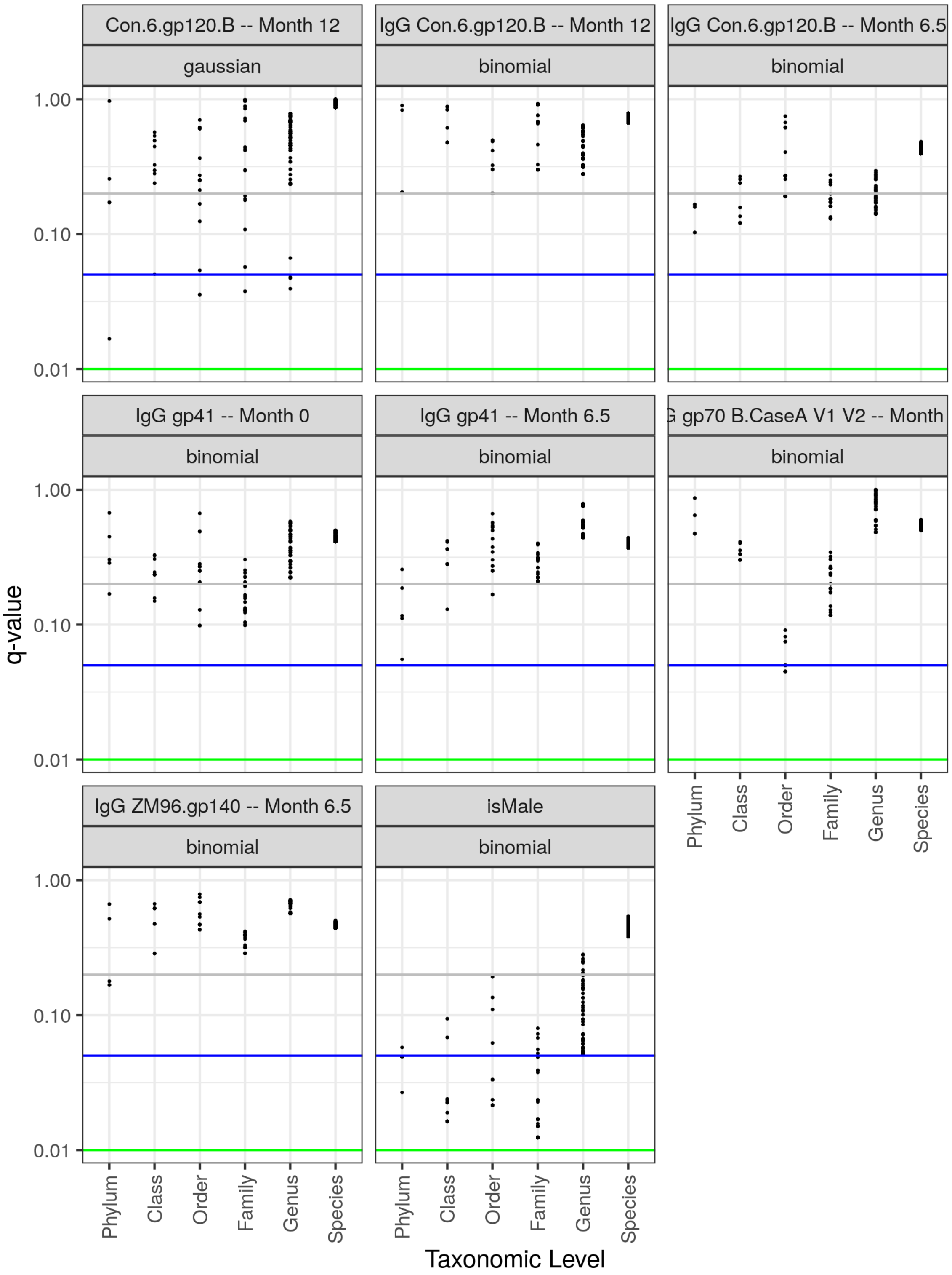

Con.6.gp120.B -- Month 12
gaussian
IgG Con.6.gp120.B -- Month 12
binomial
IgG Con.6.gp120.B -- Month 6.5
binomial
IgG gp41 -- Month 0
binomial
IgG gp41 -- Month 6.5
binomial
G gp70 B.CaseA V1 V2 -- Month
binomial
IgG ZM96.gp140 -- Month 6.5
binomial
isMale
binomial
q-value
1.00
0.10
0.01
Phylum
Class
Order
Family
Genus
Species
Taxonomic Level

S5 Fig. Statistical significance of regressions of taxa agglomerated at a range of taxonomic levels, against each antibody found to relate to community structure in Tables 1 and S1. We report both **A** *p*-values and **B** False discovery rates for each. False discovery rates are calculated from *p*-values at each antibody-taxonomic level combination. Horizontal lines indicate significance thresholds of 0.2 (gray), 0.05 (blue) and 0.01 (green).
