# Supplementary material for: Human gut microbiota is associated with HIV-reactive immunoglobulin at baseline and following HIV vaccination
Source: PLoS One. 2019 Dec 23;14(12):e0225622. doi: 10.1371/journal.pone.0225622 (PMC6927600; doi:10.1371/journal.pone.0225622)
Supplement: S6 Fig — Coefficients (y-axis) of general linear models relating family level taxa (x-axis) to antibody concentrations for which there was at least one statistically significant hit in S5 Fig. Error bars represent two standard errors of the coefficient. Colors and shapes indicate whether models have p-values < 0.05 and FDR < 0.2, respectively. Only taxa involved in at least one statistically significant association are shown. These same families are indicated with dots in Fig 4 when they are statistically significant. (PDF) [file pone.0225622.s006.pdf]

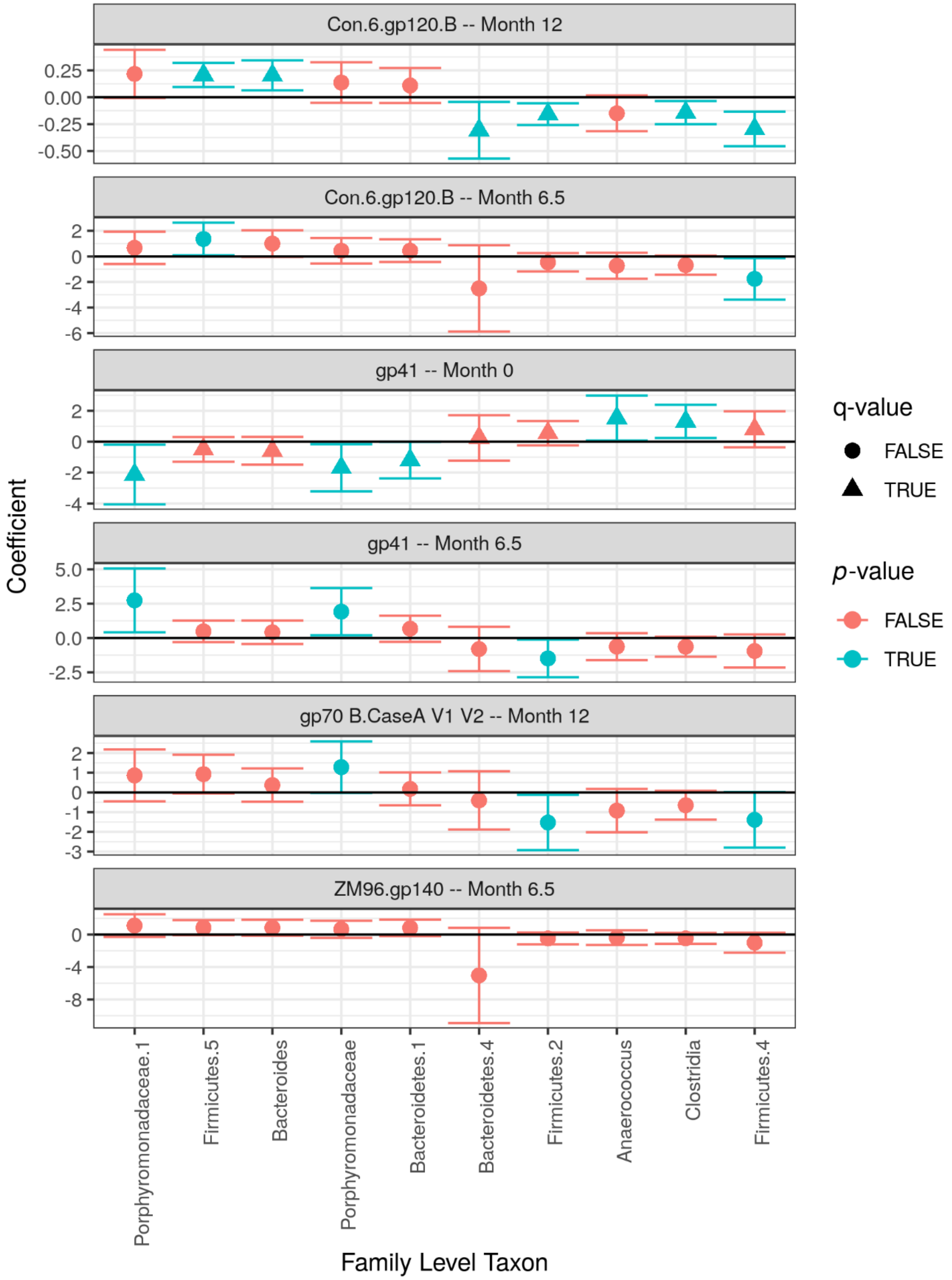

Figure S6. Coefficients (y-axis) of general linear models relating family level taxa (x-axis) to antibody concentrations for which there was at least one statistically significant hit in S5 Fig. Error bars represent

two standard errors of the coefficient. Colors and shapes indicate whether models have *p*-values < 0.05 and FDR < 0.2, respectively. Only taxa involved in at least one statistically significant association are shown. These same families are indicated with dots in Fig 4 when they are statistically significant.
