# Supplementary material for: Human gut microbiota is associated with HIV-reactive immunoglobulin at baseline and following HIV vaccination
Source: PLoS One. 2019 Dec 23;14(12):e0225622. doi: 10.1371/journal.pone.0225622 (PMC6927600; doi:10.1371/journal.pone.0225622)
Supplement: S1 Table — Numbers of participants per vaccine treatment group (columns), and time points from which their microbiota were collected for this study (rows). Treatment codes, T1-T4, are described in Section 1.1. (PDF) [file pone.0225622.s007.pdf]

Table S1. Numbers of participants per vaccine treatment group (columns), and time points from which their microbiota were collected for this study (rows). Treatment codes, T1-T4, are described in Section 1.1.

	**Treatment**				
**Month**	**T1**	**T2**	**T3**	**T4**	**total**
**0**	3	0	0	0	*3*
**6.5**	4	2	5	0	*11*
**12**	0	1	1	5	*7*
**total**	*7*	*3*	*6*	*5*	***21***
