# Supplementary material for: Human gut microbiota is associated with HIV-reactive immunoglobulin at baseline and following HIV vaccination
Source: PLoS One. 2019 Dec 23;14(12):e0225622. doi: 10.1371/journal.pone.0225622 (PMC6927600; doi:10.1371/journal.pone.0225622)
Supplement: S2 Table — Significance values of kernel regression tests, as well as significance values and slope coefficients of gaussian general linear models. This table mirrors Table 1, with the exception that here, antibody concentrations are treated as continuous, box-cox transformed variables, rather than binomial, median split variables. Kernel regressions ask whether participants that both have high or low concentrations of the antibody or T cell pool of interest have more similar microbiota (as measured by weighted UniFrac) than participants with dissimilar concentrations of that variable of interest. GLMs ask whether the weighted UniFrac axis 1 (MDS1) scores (Fig 1) of the participants are statistically related the variables of interest. Coefficients show the strength and direction of the association. R2 indicates Nagelkerke pseudo R2. p-values are calculated by permutation (Kernel regression) and directly (weighted Unifrac Regression). BH-FDR values are calculated from the p-values using the bioconductor q-value package. Yellow highlighting indicates statistically significant p (<0.05) and FDR (<0.20) values. Regression coefficients corresponding kernel p < 0.05 and FDR < 0.2, values are color-coded according to their sign, red = negative, there are no positive coefficients meeting our significance thresholds. (PDF) [file pone.0225622.s008.pdf]

S2 Table. Significance values of kernel regression tests, as well as significance values and slope coefficients of gaussian general linear models. This table mirrors Table 1, with the exception that here, antibody concentrations are treated as continuous, box-cox transformed variables, rather than binomial, median split variables. Kernel regressions ask whether participants that both have high or low concentrations of the antibody or T cell pool of interest have more similar microbiota (as measured by weighted UniFrac) than participants with dissimilar concentrations of that variable of interest. GLMs ask whether the weighted UniFrac axis 1 (MDS1) scores (Figure 1) of the participants are statistically related the variables of interest. Coefficients show the strength and direction of the association. R^2 indicates Nagelkerke pseudo R^2. *p*-values are calculated by permutation (Kernel regression) and directly (weighted Unifrac Regression). BH-FDR values are calculated from the *p*-values using the bioconductor q-value package. Yellow highlighting indicates statistically significant *p* (<0.05) and FDR (<0.20) values. Regression coefficients corresponding kernel $p < 0.05$ and FDR < *0.2,* values are color-coded according to their sign, red = negative, there are no positive coefficients meeting our significance thresholds.

			**Kernel**		**MDS**			
**Type**	**Antigen**	**Month**	**P**	**FDR**	**P**	**FDR**	**R^2**	**Coef**
CD4+	Any ENV PTEG	6.5	0.602	0.803	0.597	0.344	0.015	-0.196
		12	0.185	0.492	0.329	0.248	0.054	-0.356
IgA	gp41	0	0.989	0.989	0.633	0.344	0.013	0.177
		6.5	0.411	0.636	0.601	0.344	0.015	-0.194
		12	0.651	0.813	0.504	0.329	0.026	0.252
	p24	0	0.954	0.989	0.433	0.303	0.033	0.289
		6.5	0.976	0.989	0.857	0.441	0.002	-0.067
		12	0.575	0.803	0.309	0.248	0.058	-0.377
IgG	Con.6.gp120.B	6.5	0.073	0.366	**0.032**	**0.093**	0.208	-0.721
		12	**0.001**	**0.014**	**0.000**	**0.000**	0.530	***-1.150***
	gp41	0	0.221	0.492	**0.038**	**0.093**	0.197	0.701
		6.5	0.219	0.492	0.080	**0.102**	0.148	-0.608
		12	0.263	0.514	0.173	**0.169**	0.095	-0.486
	gp70 B.CaseA V1-V2	6.5	0.282	0.514	0.206	**0.183**	0.083	-0.454
		12	0.111	0.444	0.083	**0.102**	0.145	-0.602
	p24	0	0.753	0.886	0.907	0.444	0.001	0.044
		6.5	**0.027**	**0.193**	0.053	**0.102**	0.184	***-0.787***
		12	0.413	0.636	0.133	**0.145**	0.113	-0.531
	ZM96.gp140	6.5	**0.029**	**0.193**	**0.017**	**0.083**	0.246	***-0.783***
		12	0.198	0.492	0.078	**0.102**	0.150	-0.612
