# Supplementary material for: Human gut microbiota is associated with HIV-reactive immunoglobulin at baseline and following HIV vaccination
Source: PLoS One. 2019 Dec 23;14(12):e0225622. doi: 10.1371/journal.pone.0225622 (PMC6927600; doi:10.1371/journal.pone.0225622)
Supplement: S3 Table — p-values of logistic regressions between UniFrac PCoA components 1–10 (MDS1-MDS10) and the median split transformed concentrations of the antibodies described in Table 1. MDS1 is associated with several immune responses (p < 0.05, FDR < 0.2), the other MDS components are not. (PDF) [file pone.0225622.s009.pdf]

S3 Table. *p*-values of logistic regressions between UniFrac PCoA components 1-10 (MDS1-MDS10) and the median split transformed concentrations of the antibodies described in Table 1. MDS1 is associated with several immune responses ($p < 0.05$, FDR < 0.2), the other MDS components are not.

| Type | Antigen | Month | MDS1 | MDS2 | MDS3 | MDS4 | MDS5 | MDS6 | MDS7 | MDS8 | MDS9 | MDS10 |
|---|---|---|---|---|---|---|---|---|---|---|---|---|
| IgA | gp41 | 0.0 | 0.658 | 0.579 | 0.403 | 0.829 | 0.700 | 0.656 | 0.210 | 0.432 | 0.041 | 0.840 |
| | | 6.5 | 0.175 | 0.371 | 0.755 | 0.556 | 0.122 | 0.196 | 0.428 | 0.377 | 0.905 | 0.935 |
| | | 12.0 | 0.871 | 0.207 | 0.440 | 0.390 | 0.819 | 0.751 | 0.610 | 0.111 | 0.104 | 0.166 |
| | p24 | 0.0 | 0.325 | 0.690 | 0.570 | 0.952 | 0.806 | 0.571 | 0.141 | 0.940 | 0.981 | 0.531 |
| | | 6.5 | 0.678 | 0.297 | 0.677 | 0.310 | 0.640 | 0.622 | 0.934 | 0.214 | 0.891 | 0.341 |
| | | 12.0 | 0.390 | 0.683 | 0.259 | 0.447 | 0.079 | 0.501 | 0.608 | 0.156 | 0.630 | 0.129 |
| IgG | Con.6.gp120.B | 6.5 | **0.017** | 0.615 | 0.366 | 0.690 | 0.328 | 0.493 | 0.859 | 0.685 | 0.724 | 0.208 |
| | | 12.0 | **0.030** | 0.982 | 0.350 | 0.674 | 0.965 | 0.559 | 0.512 | 0.362 | 0.753 | 0.260 |
| | gp41 | 0.0 | **0.040** | 0.910 | 0.369 | 0.810 | 0.375 | 0.209 | 0.588 | 0.631 | 0.873 | 0.450 |
| | | 6.5 | 0.058 | 0.266 | 0.268 | 0.918 | 0.714 | 0.144 | 0.670 | 0.417 | 0.179 | 0.666 |
| | | 12.0 | 0.806 | 0.328 | 0.412 | 0.189 | 0.961 | 0.261 | 0.747 | 0.051 | 0.424 | 0.050 |
| | gp70 B.CaseA V1-V2 | 6.5 | 0.602 | 0.890 | 0.289 | 0.592 | 0.397 | 0.603 | 0.422 | 0.164 | 0.042 | 0.965 |
| | | 12.0 | **0.036** | 0.698 | 0.729 | 0.631 | 0.216 | 0.307 | 0.964 | 0.179 | 0.440 | 0.229 |
| | p24 | 0.0 | 0.428 | 0.238 | 0.925 | 0.182 | 0.070 | 0.060 | 0.664 | 0.616 | 0.479 | 0.401 |
| | | 6.5 | 0.397 | 0.857 | 0.271 | 0.351 | 0.429 | 0.096 | 0.174 | 0.726 | 0.396 | 0.078 |
| | | 12.0 | 0.176 | 0.735 | 0.247 | 0.680 | 0.856 | 0.373 | 0.035 | 0.520 | 0.735 | 0.121 |
| | ZM96.gp140 | 6.5 | **0.030** | 0.563 | 0.217 | 0.964 | 0.203 | 0.923 | 0.878 | 0.864 | 0.100 | 0.408 |
| | | 12.0 | 0.186 | 0.330 | 0.304 | 0.734 | 0.757 | 0.354 | 0.558 | 0.363 | 0.020 | 0.126 |
| CD4+ | Any ENV PTEG | 6.5 | 0.219 | 0.619 | 0.750 | 0.230 | 0.140 | 0.088 | 0.574 | 0.096 | 0.634 | 0.285 |
| | | 12.0 | 0.231 | 0.608 | 0.491 | 0.266 | 0.061 | 0.922 | 0.658 | 0.154 | 0.774 | 0.686 |
