# Supplementary material for: Human gut microbiota is associated with HIV-reactive immunoglobulin at baseline and following HIV vaccination
Source: PLoS One. 2019 Dec 23;14(12):e0225622. doi: 10.1371/journal.pone.0225622 (PMC6927600; doi:10.1371/journal.pone.0225622)
Supplement: S4 Table — Coefficients, R2 values and significance values of the relationship between alpha diversity and box-cox transformed and then z-normalized immunogenicity responses calculated using the betta function in breakaway. Highlighting and FDR calculations were applied as described in Table 1. R2 values were calculated from the pearson correlation coefficient between alpha diversity and median split immunogenicity values. (PDF) [file pone.0225622.s010.pdf]

S4 Table. Coefficients, R^2 values and significance values of the relationship between alpha diversity and box-cox transformed and then z-normalized immunogenicity responses calculated using the betta function in breakaway. Highlighting and FDR calculations were applied as described in Table 1. R^2 values were calculated from the pearson correlation coefficient between alpha diversity and median split immunogenicity values.

Type	Antigen	Month	Coef	R^2	P	FDR
CD4+	Any ENV PTEG	6.5	**30.50**	0.13	**0.016**	**0.064**
		12.0	27.49	9.9e-04	0.094	0.235
IgA	gp41	0.0	-6.51	0.03	0.718	0.818
		6.5	2.15	0.06	0.890	0.890
		12.0	**32.88**	0.09	**0.045**	**0.150**
	p24	0.0	-6.77	2.8e-04	0.714	0.818
		6.5	15.87	0.03	0.374	0.680
		12.0	25.20	0.06	0.226	0.452
IgG	Con.6.gp120.B	6.5	6.48	2.4e-06	0.706	0.818
		12.0	5.36	7.9e-05	0.736	0.818
	gp41	0.0	10.14	1.2e-04	0.510	0.774
		6.5	**29.66**	0.18	**0.002**	**0.010**
		12.0	**31.71**	0.17	**< 0.001**	**<0.001**
	gp70 B.CaseA V1-V2	6.5	3.24	2.1e-03	0.861	0.890
		12.0	**36.15**	0.03	**< 0.001**	**<0.001**
	p24	0.0	-14.39	0.1	0.421	0.702
		6.5	21.71	0.14	0.175	0.389
		12.0	31.96	0.21	0.060	**0.171**
	ZM96.gp140	6.5	8.40	4.7e-04	0.542	0.774
		12.0	**32.21**	0.09	**< 0.001**	**<0.001**
