# Supplementary material for: Human gut microbiota is associated with HIV-reactive immunoglobulin at baseline and following HIV vaccination
Source: PLoS One. 2019 Dec 23;14(12):e0225622. doi: 10.1371/journal.pone.0225622 (PMC6927600; doi:10.1371/journal.pone.0225622)
Supplement: S1 Methods — (PDF) [file pone.0225622.s011.pdf]

1 Supplemental methods

1.1 Vaccine Details

NYVAC is a combination of two attenuated vaccinia viruses, one NYVAC-HIV-PT1 contained DNA expressing HIV clade C ZM96 gp140 and the other NYVAC-HIV-PT4 contained DNA expressing clade C ZM96 Gag, ZM96 gp120 and a CN54 Pol-Nef fusion construct and two clade C gp120 proteins with MF59 adjuvant (Tartaglia et al., 1992). NYVAC was administered intramuscularly as 1mL, each at a concentration of 5 x 10^6 PFU/mL, of NYVAC-HIV-PT1 and NYVAC-HIV-PT4. The trivalent bare DNA plasmid, administered at a volume of 1mL and concentration 4mg/mL, also expressed the clade C ZM96 Gag, ZM96 gp120 and a CN54 Pol-Nef fusion construct. It was developed at the Dale and Betty Bumpers Vaccine Research Center (VRC), National Institute of Allergy and Infectious Diseases (NIAID), National Institutes of Health (NIH) (Bethesda, MD, USA). AIDSVAX®, is a bivalent gp120 glycoprotein, containing sequences of the MN and A244 HIV-1 strains, currently developed by Global Solutions for Infectious Diseases. It is administered intramuscularly at a volume of 1ml and concentration of 300 mcg/ml along with 600mcg Alum/ml Aluminum hydroxide gel adjuvant.

1.2 Microbial DNA Extraction

Rectal secretion samples were extracted along with the antibody samples from rectal wecks as described previously [3]. Briefly, weck cell sponges rinsed three times with extraction buffer (1X PBS (Invitrogen 10010-023), Protease inhibitor (Sigma 539131), and 0.25% bovine serum albumen (Sigma A8412)) which was removed from the filter after each rinse by centrifugation on a spin-X filter at 16000 x g. The MoBio Bacteremia kit was used to extract DNA from the rinse solution.

1.3 Processing of sequence data

Microbial 16S V3-V4 amplicon data were processed and analyzed using a series of BASH scripts and Python Jupyter Notebooks containing R code that are available at (https://github.com/cramjaco/Nyvac_096_Microbiome). Briefly, samples were demultiplexed and barcodes and primers removed in QIIME1. Sequence variant (SV) assignment was carried out using an adaptation of the DADA2 pipeline for 454 data (https://benjjneb.github.io/dada2/faq.html#can-i-use-dada2-with-my-454-or-ion-torrent-data, see Supplemental methods). SVs were named with DADA2's taxonomic identification functions and a phylogenetic tree of SVs from within all samples was generated in the R environment (v. 3.4.1) using the *phangorn* package [4]. For the purposes of this analysis, we removed 31 SVs that were unidentified to the Phylum level, 7 SVs from phyla that were found in the data set fewer than 20 times each (Verrumicrobia, Tenericutes, Elusimicrobia and Synergistetes), and 386 SVs that were present in fewer than 10% of the samples.

To test the association of microbial subpopulations with immunogenicity and vaccine response, we clustered the 16S SVs at multiple levels of phylogenetic relatedness. Because of the inconsistencies observed with taxonomic classification algorithms [5], we avoided a taxonomic approach to phylogenetic clustering, instead using the phylogenetic diversity based on multiple sequence alignment of the complete set of SVs in this study. We created a set of clusters with the goal of approximating the degree of granularity that would be achieved by clustering at the commonly accepted phylogenetic levels (e.g. Phylum, Class), while allowing our groups to be independent of any taxonomic classification algorithm.

Operationally, phylogenetic clustering was performed by (1) identifying the number of unique taxa that were predicted to be present at each taxonomic level (e.g. Phylum, Class) according to DADA2's implementation of RDP's naive Bayesian classifier [6], (2) performing agglomerative clustering on the phylogenetic tree of SV sequences to create the number of groups identified at each of those taxonomic

levels, and (3) naming the resulting phylogenetic groups according to the highest taxonomic level shared by SVs within that group.

1.4 Global Tests

In two versions of this analysis, immunological variables were "median-split" and treated as a binary variable, and secondarily box-cox transformed. This same kernel regression method was used to compare Jensen-Shannon divergence to each of the BAMA measurements. To perform this analysis, Jensen-Shannon distance matrix was derived from community structure data that had been agglomerated to each taxonomic level (Supplement Section 1.3). MiRKAT was then provided a list of kernels, each one representing distance matrices from communities aggregated to different levels. Then for each variable of interest, MiRKAT calculated kernel regression *p*-values for each level of taxonomic agglomeration. For the of Jensen-Shannon kernel regression tests of different taxonomic agglomerations, MiRKAT calculated an omnibus *p*-value, which determines whether a family of related tests shows significance overall. We performed logistic regression of MDS1, the major component of weighted UniFrac variability, against both the median split transformed values (respectively) of each IgG and IgA. As a secondary analysis, we performed linear regression of MDS1 against the box-cox transformed measurements. For all analyses, we report coefficients and McFadden's R^2 values for the linear and logistic regression approaches, but not for the kernel regression approach, which reports *p*-values but not coefficients.

Species richness was estimated with the breakaway package. We used breakaway's betta function to examine the relationship between richness and median split and box-cox transformed immunogenicity measurements. We also examined whether richness was associated with unifrac distance, again using the MiRKAT package; or to MDS1, using breakaway's betta function.

1.5 Local Regression Tests

For each immunologic variable that was found to be statistically significant under the global test, we performed (at each agglomeration level) logistic regression of each taxon's centered log-ratio (clr) transformed relative abundance against median split immunological measurements. Multiplicity adjustment to control the false-discovery rate (FDR) was performed using the Benjamini and Holchberg method [7], but implemented by the q-value R package [1] across all taxa for each immune variable at each level of agglomeration. We identified which taxonomic agglomeration level - immunologic variable combinations were associated with some FDR < 0.2. These local associations are intended to be descriptive, and even with FDR < 0.2, we do not mean to imply that any specific association is statistically significant.

1.6 Statistical associations between Family level groups, and between Families and Immune variables:

Because the local tests identified that family level groups had more statistically significant interactions than groups at other levels (see Results Section 2.4), subsequent analysis focused on family level patterns. We report which family level taxa are associated, via logistic regression with each immunologic variable that relates to community structure via the global kernel regression tests. Gp120 binding IgG; which associated more strongly with community structure when it was treated as a discrete (Table 1), rather than a continuous (Table S2) variable; was also treated as a continuous variable, box-cox transformed, and linearly regressed against species abundances.

To understand how these local associations related to microbial community structure patterns, we investigated whether those taxa that were associated with immunological variable abundance were also associated with each other. The proportionality method [8], was used to test for statistical associations between family level groups, while accounting for compositionality of the groups. We examined whether

these co-occurring families were systematically related to relationships between the taxa and immune measurements.

1.7 References

1. Storey JD. A direct approach to false discovery rates. J R Stat Soc Ser B Stat Methodol. 2002 Aug 1;64(3):479–98.

2. Zhao N, Chen J, Carroll IM, Ringel-Kulka T, Epstein MP, Zhou H, et al. Testing in Microbiome-Profiling Studies with MiRKAT, the Microbiome Regression-Based Kernel Association Test. Am J Hum Genet. 2015 May 7;96(5):797–807.

3. Pantaleo G, Janes H, Karuna S, Grant S, Ouedraogo L. Co-administration of HIV Env protein with DNA and/or NYVAC vaccines in humans results in earlier and potent generation of anti-Env antibody responses. Lancet HIV. In Press;

4. Schliep KP. phangorn: phylogenetic analysis in R. Bioinformatics. 2011 Feb 15;27(4):592–3.

5. Golob JL, Pergam SA, Srinivasan S, Fiedler TL, Liu C, Garcia K, et al. Stool Microbiota at Neutrophil Recovery Is Predictive for Severe Acute Graft vs Host Disease After Hematopoietic Cell Transplantation. Clin Infect Dis. 2017 Nov 29;65(12):1984–91.

6. Wang Q, Garrity GM, Tiedje JM, Cole JR. Naive Bayesian classifier for rapid assignment of rRNA sequences into the new bacterial taxonomy. Appl Environ Microbiol. 2007 Aug;73(16):5261–7.

7. Benjamini Y, Hochberg Y. Controlling the False Discovery Rate: A Practical and Powerful Approach to Multiple Testing. J R Stat Soc Ser B Methodol. 1995;57(1):289–300.

8. Lovell D, Pawlowsky-Glahn V, Egozcue JJ, Marguerat S, Bähler J. Proportionality: A Valid Alternative to Correlation for Relative Data. PLOS Comput Biol. 2015 Mar 16;11(3):e1004075.
